# Analysis of lattice changes and weak inter­actions in a series of sol­vato­morphic isostructural spin-crossover com­pounds

**DOI:** 10.1107/S2053229626010740

**Published:** 2026-10-20

**Authors:** Daniel Brüx, Johannes Bartusel, Florian Kleemiss

**Affiliations:** a RWTH Aachen University, Institut für Anorganische Chemie, Landoltweg 1a, 52074 Aachen, Germany; University of Oxford, United Kingdom

**Keywords:** crystal structure, spin-crossover, weak inter­actions, NCI, Hirshfeld surface

## Abstract

Five new solvate forms of the com­plex bis­(thio­cyanato-κ*N*)[tris­(pyridin-2-ylmeth­yl)amine-κ^4^*N*,*N*′,*N*′′,*N*′′′]iron(II), [Fe(NCS)_2_(C_18_H_18_N_4_)] or [Fe(NCS)_2_(tpa)], a known spin-crossover com­pound, are reported. Among the incorporated solvents are all four phenyl monohalides (fluoro-, chloro-, bromo- and iodo­benzene) and pyridine. Through variable-tem­per­a­ture single-crystal X-ray diffraction, we characterized the transition by means of lattice parameters, Fe—N bond lengths and distortion of the coordination sphere. The structures remain isostructural across the com­pound series, but exhibit distinct transition tem­per­a­tures indicating a solvate-dependent behaviour. Hirshfeld surface, noncovalent inter­action analyses and energy frameworks provide a more detailed view of how the structural response affects the inter­molecular inter­action network. In particular, the tem­per­a­ture dependence of the dominant pairwise inter­action along the crystallographic *b* direction closely mirrors the evolution of the *b*-axis lattice parameter, suggesting that this inter­action plays a key role in directing the structural response to spin crossover. Our com­parison demonstrates that different transition behaviour is not caused by one fundamental change in the packing architecture but rather by subtle solvate-dependent changes.

## Abstract 2

Five new sol­vato­morphic spin-crossover com­pounds were crystallized and structurally characterized. Variable-tem­per­a­ture X-ray diffraction, Hirshfeld surface analysis and NCI plots shed light on the role of weak inter­actions.

## Introduction

Changes in an external stimulus, such as tem­per­a­ture or pressure, can induce spin-state switching in metal com­plexes, particularly at iron(II) centres. The resulting spin crossover (SCO) has been well studied in countless solid samples, including exhaustive crystallographic analyses at the mol­ecular level (Brüx *et al.*, 2026 ▸; Guionneau, 2014 ▸; Halcrow, 2011 ▸; Collet & Guionneau, 2018 ▸; Guionneau *et al.*, 2004 ▸). The, to some extent, abrupt distortion experienced by the com­plexes during the transition is recognizable in an uneven thermal evolution of unit-cell metrics and inter­atomic dis­tances. By variation in ligand designs or counter-ions, the SCO phenomenon has been studied in terms of mol­ecular inter­actions and crystal packing (Halcrow *et al.*, 2019 ▸; Fang *et al.*, 2024 ▸; Molnár *et al.*, 2019 ▸; Pfaffeneder *et al.*, 2011 ▸). Numerous results in this context showed that even subtle variations, like the exchange of solvent in the crystal structure, can drastically change the transition behaviour (Chernyshov *et al.*, 2003 ▸; Hostettler *et al.*, 2004 ▸; Bartel *et al.*, 2007 ▸; Liu *et al.*, 2025 ▸). Remarkably, the thio­cyanate iron(II)–tpa com­plex [tpa is tris­(pyridin-2-ylmeth­yl)amine] (Højland *et al.*, 1983 ▸) is known to have several polymorphs and sol­vato­morphs, which are SCO active when subjected to tem­per­a­ture changes. Since the elucidation of its first crystal structure (Li *et al.*, 2010 ▸), four polymorphs (Wei *et al.*, 2011*a* ▸) and seven sol­vato­morphs (Wei *et al.*, 2011*b* ▸) of this com­pound have been reported up to now (Fig. 1 ▸). They exhibit varying transition behaviour depending on the solvate present. Among the different sol­vato­morphs, only a few are isostructural when the solvate mol­ecules are very similar (*e.g.* MeOH and EtOH).

Herein, we report the structures of five new solvate forms of bis­(thio­cyanato-κ*N*)[tris­(pyridin-2-ylmeth­yl)amine-κ^4^*N*,*N*′,*N*′′,*N*′′′]iron(II), **1**, which all crystallize in the same space group (*P*2_1_/*n*) with similar lattice constants (see the supporting information). They can thereby be considered as isostructural (Lima-de-Faria *et al.*, 1990 ▸). SCO com­pounds, which mimic each other in space-group symmetry and packing, naturally arise when one tries to design SCO structures that are closely alike to analyse their (yet different) transition behaviour. Often found examples are isostructures induced by anion exchange (Hibbs *et al.*, 2003 ▸; Wiehl, 1993 ▸), partial occupation of the iron position by another metal (Mueller *et al.*, 1983 ▸), or solvate exchanges (Białońska & Bronisz, 2010 ▸; Birchall *et al.*, 2023 ▸). Yet, with regard to what is already reported and together with our findings, com­pound **1** appears to be a true wellspring of sol­vato­morphs and polymorphs, and, to the best of our know­ledge, we cannot name another SCO com­pound pos­sessing more solvate forms.

The focus of this work is aimed at investigating the changes that the crystal packing exhibits in response to varying tem­per­a­tures. Especially as the weak inter­molecular inter­actions determine the transition behaviour of SCO com­pounds (Halcrow, 2011 ▸), in-depth analysis of short contacts has proven to reveal the most directing packing features (Li *et al.*, 2010 ▸; Sheu *et al.*, 2008 ▸; Pfaffeneder *et al.*, 2011 ▸). While it is the current scientific consensus that crystal packing guides the spin transition, short inter­action criteria that go beyond distance measures (Tan *et al.*, 2019 ▸) are rarely used for in-depth analysis of SCO com­pounds (Miyawaki *et al.*, 2020 ▸; Novikov *et al.*, 2014 ▸; Díaz-Torres *et al.*, 2022 ▸; Tailleur *et al.*, 2019 ▸; Reeves *et al.*, 2021 ▸). The cause may be that already small differences in any structural aspect can change the SCO behaviour, making an estimate of influences hard to generalize (Díaz-Torres *et al.*, 2022 ▸; Real *et al.*, 2003 ▸; Takahashi *et al.*, 2010 ▸). So, with our series of iron(II)–tpa com­plexes, which are very similar in their crystal packing, we want to gain structural insights through variable-tem­per­a­ture single-crystal X-ray diffraction and further rationalize inter­molecular inter­actions. For the latter, we use com­putational methods like Hirshfeld surfaces (Spackman & McKinnon, 2002 ▸; McKinnon *et al.*, 2007 ▸), energy frameworks (Mackenzie *et al.*, 2017 ▸) and NCI plots (Contreras-García *et al.*, 2011 ▸). The experimentally determined geometric inputs for the named methods are enhanced through Hirshfeld Atom Refinement (HAR) (Capelli *et al.*, 2014 ▸), which was shown to improve structural quality and was made readily available (Kleemiss *et al.*, 2021*a* ▸).

## Experimental

### Synthesis and crystallization

#### Synthesis of the phenyl halide solvates of com­pound 1

A general procedure for the synthesis of suitable single crystals for diffraction analysis, where all solvents were degassed prior to use, was employed. A solution of tpa (70 µmol, 1 equiv.) dissolved in the appropriate phenyl halide (1 ml) was placed at the bottom of a Schlenk tube. Above the ligand solution, a mixture of acetone and phenyl halide (2 ml, 1:1 *v*:*v*) was carefully layered. The top layer contains [Fe(NCS)_2_(py)_4_] (70 µmol, 1 equiv.) in acetone. When left un­dis­turbed, large single crystals of **1**·FPh and **1**·BrPh were ob­tained after 1–3 d. Crystals of **1**·ClPh and **1**·IPh were obtained after the solutions were slowly concentrated by a steady flow of nitro­gen.

#### Synthesis of the pyridine solvate of com­pound 1

Bulk material of **1** was prepared as reported previously (Yu *et al.*, 1994 ▸). Recrystallization from pyridine yields crystals of **1**·py when the solution was left undisturbed in a fridge.

### Refinement

Crystal data, data collection and structure refinement details for all determinations are summarized in the supporting information. For each com­pound, a suitable crystal was chosen and a tem­per­a­ture series of diffraction experiments was measured on an XtaLAB Synergy R diffractometer equipped with a HyPix-Arc 100 detector and Mo *K*α radiation. The tem­per­a­ture was controlled by an Oxford Cryostream 700 and the data series was collected in cooling mode only. Data integration, reduction, scaling and Gaussian absorption correction were performed in *CrysAlis PRO* (Rigaku OD, 2025 ▸). The structures were solved using *SHELXT* (Sheldrick, 2015 ▸) and further refined with *olex2.refine* within the *OLEX2* software suite (Dolomanov *et al.*, 2009 ▸; Bourhis *et al.*, 2015 ▸). Initially, the H-atom positions were kept fixed at idealized positions. Besides **1**·py, all structures exhibit disorder in the high-tem­per­a­ture phase. The disorder only affects the solvate mol­ecules, resulting in different orientations of the halide atom. While chloro- and bromo­benzene split into two parts, with occupancies refined freely, fluoro­benzene was modelled as three parts, with fixed occupancies to ensure convergence during refinement. Iodo­benzene showed a positional disorder to a great extent, which was not resolvable by explicit modelling, so a solvent mask was applied (SQUEEZE; Spek, 2015 ▸). Each final model was subjected to an iterative Hirshfeld Atom Refinement using *NoSpherA2* (Kleemiss *et al.*, 2021*a* ▸). Prior to the partitioning of the electron density, the calculation of the wavefunction was performed using *ORCA* (Version 6.0; Neese, 2012 ▸; Neese, 2023 ▸; Neese *et al.*, 2020 ▸), applying the r^2^SCAN/def2-TZVP (Furness *et al.*, 2020 ▸; Weigend & Ahlrichs, 2005 ▸) level of theory, including a water CPCM (Conductor-like Polarizable Continuum Model) model (Barone & Cossi, 1998 ▸; Brüx *et al.*, 2025 ▸). Additional crystallographic information is listed in Section 2 of the supporting information.

### Computational methods

The Hirshfeld surfaces were generated using *CrystalExplorer17* (Spackman *et al.*, 2021 ▸). For the disordered high-tem­per­a­ture phases, only the major part was included in the analysis. The normalized contact distance (*d*_norm_) surface maps are visualized at a scale between −0.1 and 1.25 a.u. Corresponding fingerprint plots are shown in the supporting information (Section 5). The pairwise energies included com­plete mol­ecular units within a radius of 3.8 Å and were calculated using the CE-B3LYP model (Mackenzie *et al.*, 2017 ▸).

Noncovalent inter­action indicators (Contreras-García *et al.*, 2011 ▸) were generated by growing an appropriate number of neighbouring mol­ecules within *OLEX2* and saving two separate .xyz files, one containing the central unit and the other containing selected surrounding mol­ecules. Based on this input, a modified version of *NoSpherA2* (Kleemiss *et al.*, 2021*b* ▸) was used to calculate the electron density and the reduced density gradient (RDG) based on Thakkar’s spherical densities (Kleemiss *et al.*, 2024 ▸). The outputs were visualized in *VMD* (Humphrey *et al.*, 1996 ▸). Corresponding scatterplots of the RDG *versus* sign(λ)ρ are given in Section 4 of the supporting information.

## Results

Crystals of the reported com­pounds were obtained through the approach described above and can be stored in their mother solution for months. Yet, when they are removed from the solution for crystal picking, a loss of crystallinity occurs within minutes. A sample of **1**·py was analysed as a representative example by powder X-ray diffraction, confirming the weak diffracting nature of the bulk sample (see Fig. S5 in the supporting information). In all cases, the freshly picked yellow-coloured crystallites were suitable for in-house single-crystal analysis. It is advisable to mount the crystal at tem­per­a­tures above 200 K, since otherwise the abrupt lattice contraction during the spin transition leaves the crystal shattered and hinders proper data collection (see Section 1 in the supporting information). When the samples were cooled in a controlled manner (*e.g.* at a rate of 25 K h^−1^), suitable data could be collected even below the transition tem­per­a­ture.

### Crystal packing

The abrupt changes in the crystal lattice were in no case accom­panied by a change in crystal symmetry or space group, and both the high- and low-tem­per­a­ture phases crystallize in the monoclinic space group *P*2_1_/*n* with *Z*′ = 1, including a solvate mol­ecule in the asymmetric unit. The iron(II) cation is coordinated in an octa­hedral fashion, without involving the solvate in the coordination sphere. Within the unit cell, the solvate mol­ecules are always arranged at the same position along the twofold axes separated by *b*/2 [Figs. 2 ▸(*a*) and 2(*d*)]. The iron com­plex units follow this direction by a zigzag chain along the screw axes, where adjacent pairs were connected by π–π contacts in addition to nonclassical hy­dro­gen bonding (Desiraju, 1991 ▸) involving C—H⋯S contacts [Fig. 2 ▸(*b*)]. The latter involves the methyl­ene group of tpa, which was also described as a packing-directing feature in the solvent-free form of **1**, which also retains this zigzag motif along the *b* axis (Li *et al.*, 2010 ▸). Two staggered inversion-related com­plex units are also involved in a sandwich-like π-stacking that includes the solvate mol­ecules in the form solvate⋯**1**⋯**1**⋯solvate [Fig. 2 ▸(*c*)] along the cell diagonals.

### Disorder of the solvate

During the tem­per­a­ture series, the pyridine solvent mol­ecule retains its orientation (see Fig. S24) without the N atom being involved in any stronger inter­molecular inter­action, which could explain this preferred orientation. Besides **1**·py, all phenylic solvate mol­ecules exhibit a disorder with respect to the direction in which the halogen atom points. In most cases, the superposition was modelled through a rotational disorder along the twofold screw axis. In all structures, the disorder resolved upon entering the low-spin (LS) state, as signified by the absence of significant residual density in minor parts that had previously been occupied by a small fraction (Fig. 3 ▸). The orientation of the phenyl halides seems to be influenced by adjacent sulfur or halide substituents in both the disordered high-tem­per­a­ture (HT) and low-tem­per­a­ture (LT) phases, indicating a directive role of inter­halogen contacts (Desiraju & Parthasarathy, 1989 ▸). For the iodine variant, a severe displacement of the solvate over several positions hindered the modelling of the disorder and the HT datasets were subjected to *PLATON* SQUEEZE (Spek, 2015 ▸; Spek, 2020 ▸) to give a reasonable structure. The Cl- and Br-containing com­ponents show a similar disorder in which the minor fraction of the solvate is slightly rotated (∼29° for Cl and ∼46° for Br) with respect to the major fraction. At 270 K, the highest evaluated tem­per­a­ture, the (minor) part has an occupancy of 41.8 (3)% for Br and 50.5 (4)% for Cl, which decreases to 34.2 (3)% for Br and to 27.8 (2)% for Cl at the final tem­per­a­ture before the transition. Measures of inter­molecular distances for the solvates are displayed in Fig. 3 ▸ and show that the disordered parts prefer different short contacts. These mainly include the thio­cyanate S atom of an adjacent com­plex, the halide of a neighbouring solvate and the above-mentioned solvate⋯**1** π-stacking within the asymmetric unit. In **1**·ClPh, the major part prefers Cl⋯Cl contacts over Cl⋯S contacts, while the minor part shows the opposite behaviour. The mentioned inter­atomic distances fall into the regime of halogen or inter­halogen contacts (Grineva, 2023 ▸) and with decreasing tem­per­a­ture, the conformation having the shorter Cl⋯Cl distance [3.5148 (17) Å; see Fig. 3 ▸(*b*)] gets more populated, reducing the presence of the shorter Cl⋯S contact [3.307 (4) Å]. Compound **1**·BrPh shows the opposite behaviour, favouring the Br⋯S contact as the tem­per­a­ture decreases [Fig. 3 ▸(*c*)]. Less difference in the π-stacking can be observed between the disordered parts in both **1**·ClPh and **1**·BrPh. The fluoro­benzene solvate is expected to have the weakest affinity to form an F⋯F inter­halogen bond (Jelsch *et al.*, 2015 ▸), which is reflected in the distance of 4.21 (3) Å in the HT phase [Fig. 3 ▸(*a*)]. The disordered part having this F⋯F contact is one of three parts being included in the disorder model of the HT phase, and it is also the least occupied. The other parts show close contacts towards the thio­cyanate S atom of different adjacent com­plex units, ranging around 3.2 Å. Only one F⋯S contact remains after the transition into the LT phase, a conformational difference unique to the fluoro­benzene solvate. The Cl-, Br- and I-containing com­pounds also result in a disorder-free model, averaging the initially split solvate position and closely resemble each other. The *X*⋯S distances follow the trend Cl⋯S < Br⋯S < I⋯S, while the *X*⋯*X* distance follows an I⋯I < Br⋯Br < Cl⋯Cl order. The π–π plane-centroid-to-centroid distance also increases from F to I. In particular, the contrast between a (partially) severely disordered HT phase and a fully ordered LT phase indicates the significant lattice changes that the com­pounds undergo, thereby forcing the solvates to adopt a single conformation.

### Temperature dependence of the unit-cell parameters

The tem­per­a­ture-dependent unit-cell parameters are available for all reported com­pounds, since their single-crystal structures were evaluated at different tem­per­a­tures over a broad tem­per­a­ture inter­val (see Fig. 4 ▸). In all com­pounds, the unit-cell volume decreases upon cooling and the absolute volumes differ according to the incorporated solvent mol­ecules. The structural response associated with the spin conversion is indicated by discontinuities or changes in slope within a com­pound-specific tem­per­a­ture inter­val. Approximate transition regions are: py 150–175 K, F 150–155 K, I 155–160 K, Cl 165–170 K and Br 180–190 K. For the F-, Cl-, Br- and I-containing com­pounds, the transition into the low-spin phase is characterized by an abrupt contraction of the *a* axis, accom­panied by an elongation of the *b* axis. The *c* axis decreases moderately, with only small discontinuities near the corresponding transition tem­per­a­ture. Compound **1**·py exhibits a different response: the changes are distributed over a broader tem­per­a­ture inter­val. Pronounced variations are found in the *a* and *c* axes, whereas the *b* axis changes only slightly. The monoclinic angle *β* shows no uniform trend across the series. Compound **1**·IPh exhibits a pronounced increase in *β* near 155 K, whereas the changes for the com­pounds containing the F, Cl, Br and pyridine solvates are smaller and differ in sign. Thus, although the com­pounds retain a common structural type, incorporating different solvates modifies the tem­per­a­ture-dependent lattice response. The behaviour is consistent with previous observations, *i.e.* that solvent inclusion can strongly influence the SCO even when the principal host packing remains essentially unchanged (Cook *et al.*, 2016 ▸).

### Temperature dependence of the iron(II) coordination centre

While the unit-cell parameters describe how the crystal lattice responds collectively to the SCO, they do not directly reveal the local structural changes within the iron(II) coordination sphere, such as changes in bond length and octa­hedral distortion. Hence, a direct structural indicator is the mean Fe—N bond length (〈Fe—N〉), which increases upon population of the anti­bonding orbitals in the HS state. In addition to this expansion, the deviation of the coordination environment from an ideal octa­hedron can be qu­anti­fied using an angular distortion parameter (Collet & Guionneau, 2018 ▸) Σ with
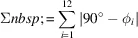
where ϕ represents the 12 N—Fe—N angles. The value of Σ = 0° corresponds to an ideal octa­hedron, whereas larger values indicate progressively stronger angular distortion of the iron coordination sphere.

The tem­per­a­ture dependence of 〈Fe—N〉 and Σ is shown in Fig. 5 ▸. At high tem­per­a­tures, the mean Fe—N distances range from approximately 2.16 to 2.18 Å across all com­pounds, consistent with a high-spin state of iron(II). Upon cooling, all com­pounds undergo a decrease to 1.96–1.98 Å, characteristic of the low-spin state. The magnitude of the bond-length change is similar throughout the series, whereas the tem­per­a­ture range and abruptness of the conversion depend on the incorporated solvent.

Compounds **1**·ClPh and **1**·BrPh display the most abrupt changes, and the Br com­pound shows the transition at the highest tem­per­a­ture near 190 K, while **1**·ClPh has the second highest transition at around 165 K. Both **1**·FPh and **1**·IPh display a less abrupt transition, shifted towards lower tem­per­a­tures at approximately 150 K. In contrast, **1**·py shows a broader and more gradual evolution, extending approximately from 145 to 190 K. The octa­hedral distortion follows the same overall trend. At high tem­per­a­tures, Σ exceeds 80°, while after the transition to the LT phase, the parameter is approximately 40° for all com­pounds. The changes in Σ occur within the same tem­per­a­ture inter­vals as the corresponding changes in the mean Fe—N distances, with the F-, Cl- and Br-containing com­pounds showing a step-like change, while **1**·IPh displays a more gradual evolution during cooling. Compound **1**·py again differs by exhibiting a broader continuous decrease in Σ, consistent with the more gradual evolution of bond length. Taken together, although the low- and high-tem­per­a­ture geometries are broadly com­parable across the series, the nature of the transition is strongly solvate dependent.

## Discussion

The different transition tem­per­a­tures and steepness observed in the variable-tem­per­a­ture structural analysis indicate that the five com­pounds exhibit varying degrees of cooperativity despite their close structural relationship. Cooperativity describes the extent to which the spin-state change at one metal centre influences the transition of neighbouring centres (Kreutzburg *et al.*, 2017 ▸; Slichter & Drickamer, 1972 ▸). Stronger or more efficiently connected mol­ecular architectures can therefore promote a more collective SCO, resulting in a sharper transition, whereas weaker coupling generally leads to a more gradual crossover. The differences observed across the series consequently motivate a closer examination of inter­molecular environments.

Representation of the mol­ecular environment within the crystal is enabled by Hirshfeld surfaces, which identify specific close inter­molecular contacts. Complementarily, NCI analysis visualizes inter­actions as isosurfaces of the reduced density gradient mapped on the electron density. The distinction between attractive, weak noncovalent and repulsive is indicated by the respective colouration of the NCI isosurface in blue, green and red regions (Tan *et al.*, 2019 ▸). Fig. 6 ▸ shows the FPh-containing structure as a representative example at two tem­per­a­tures: before the transition at 155 K [Figs. 6 ▸(*a*) and 6(*c*)] and after the transition at 150 K [Figs. 6 ▸(*b*) and 6(*d*)]. The Hirshfeld surface around the central com­plex is dominated by a small number of recurring inter­molecular contacts in both states. These include most notably the F⋯S contact, C—H⋯S inter­actions and π–π contacts between neighbouring aromatic fragments. The latter are highlighted by the NCI isosurfaces [Figs. 6 ▸(*a*) and 6(*b*)]. A com­parison of the HS and LS structures shows that the overall con­tact pattern is retained, consistent with the isostructural character of the transition, but the prominence of some contacts changes upon spin conversion. In the LS state, the con­trac­tion of the coordination sphere is accom­panied by a generally denser packing arrangement, as reflected in the more pronounced Hirshfeld surface features. The NCI isosurfaces at the relevant inter­action sites mostly agree with that but also reveal more pronounced weak C—H⋯S inter­actions towards the neighbouring com­plex depicted below (symmetry code: −*x* + 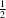, *y* + 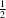, −*z* + 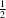). Despite being subtle, this indicates that these inter­actions accommodate or respond to the mol­ecular contraction associated with the transition. The aromatic stacking and F⋯S inter­action are com­parably unaffected and may therefore contribute less to the nature of the transition. This is consistent with previous observations that short inter­molecular contacts, including (nonclassical) hy­dro­gen bonds involving the NCS ligands, are more effective in transmitting cooperative structural changes than longer-range inter­actions, such as π–π contacts (Pfaffeneder *et al.*, 2011 ▸). Taken together, the listed tightening and changes in pre-existing inter­molecular contacts may define pathways for the propagation of structural changes through the lattice. Even though the above analysis describes **1**·FPh, it also generally accounts for the other com­pounds, for which the corresponding plots are provided in Section 3 of the supporting information. As the differences in close contacts between the HS and LS states are only marginally reflected in the corresponding fingerprint plots, they are not discussed further, but are included in the supporting information for the sake of com­pleteness (see Figs. S15–S19).

Hirshfeld surface and NCI analyses identify short contact motifs around the SCO centre, but they do not qu­antify their relative energetic contributions. Beyond this purely qualitative description of the short contacts, *CrystalExplorer* (Spackman *et al.*, 2021 ▸) pairwise inter­molecular inter­action energies were calculated for the mol­ecular pairs surrounding the central com­plex. To then visualize how the pairwise inter­actions are distributed throughout the crystal lattice, an energy framework is constructed. The calculation relies on the assumption that an inter­action energy can be expressed as the sum of its contributions, which are the electrostatic, polarization, dispersion and repulsion energy (Turner *et al.*, 2015 ▸). The total energy is then visualized as rods between the mol­ecular centres, and the rod radius indicates the strength of the inter­action. The usefulness of the method for assessing cooperativity has already been demonstrated (Reeves *et al.*, 2021 ▸). Figs. 7 ▸(*a*)–(*e*) show the energy frameworks of every com­pound before and after the transition. The chosen orientation reveals the anisotropy of the inter­action network. The strongest energies form continuous columns along the *b* direction, thereby defining a supra­molecular backbone for all structures. Thinner cylinders connecting neighbouring columns indicate weaker lateral inter­actions, which nevertheless contribute to a three-dimensionality. Also noteworthy is the energy framework of **1**·IPh [Fig. 7 ▸(*e*)], since the solvate was omitted for the calculation in this case. Thus, it shows that the *b*-dominated network persists even in the absence of the energetic contributions of the solvate. Noteworthy is also that the F-, Cl- and Br-containing com­pounds show differently pronounced tightness of the lateral connecting rods depending on the spin state. In particular, the ClPh-containing com­pound [Fig. 7 ▸(*b*)] shows fewer inter­connections in the contracted unit cell, whereas the lateral connections increase for **1**·FPh and **1**·BrPh. The strong inter­action chain along the crystallographic *b* direction suggests that structural changes associated with the spin transition are preferentially propagated along this direction. Closer inspection of the total energy framework shows that the dominant inter­action pathway along *b* is mediated by the same neighbouring com­plex–com­plex contact highlighted in the Hirshfeld surface and NCI analyses in Fig. 6 ▸.

The tem­per­a­ture-dependent pairwise inter­action energy of the dominant com­plex–com­plex contact, com­pared with the evolution of the crystallographic *b* axis, is shown in Fig. 8 ▸. For all five com­pounds, the inter­action energy follows the same overall trend as the *b* axis, confirming that the contact is directly coupled to the structural response of the system during the transition. The abrupt increase in *b* upon cooling into the LS state is marked by a destabilization of the pairwise inter­action energy, which is noteworthy, since the lattice contracts overall. This destabilization may be offset by the formation of a denser inter­action framework, as observed for the LS phases (see Fig. 7 ▸). Again, the energy calculation for **1**·IPh excluded the solvate unit, which can explain the more significant deviations com­pared to the other com­pounds. Inter­molecular inter­actions between neighbouring com­plexes which propagate approximately along the crystallographic *a* axis (symmetry code: *x* + 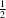, −*y* + 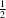, *z* + 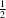) are found to be weaker. The contraction of the *a* axis during the transition is then accom­panied by an increase in inter­action strength between these neighbours (see Fig. S25), except for **1**·BrPh. However, the correlation with the *a* axis is considerably weaker com­pared to the *b* axis. No corresponding pairwise inter­action along the *c* axis has been identified. Analysis of the inter­action energies involving the incorporated solvent shows that these inter­actions are smaller than 40 kJ mol^−1^ in all cases and are therefore substanti­ally weaker than the inter­molecular inter­actions between neighbouring com­plexes (see Figs. S20–S23). In the context of the initially observed differences in transition steepness, the lattice analysis suggests that varying cooperativity of the five com­pounds originates from subtle changes in how the conserved packing motif transmits the response of the SCO centres. Even though the strong *b*-axis inter­action chain appears to represent a common pathway for structural communication, the varying solvate influences the nature of the transition remarkably, without being directly part of the strongest inter­action.

## Conclusion

In summary, variable-tem­per­a­ture single-crystal X-ray diffraction reveals that the five solvate-containing SCO com­pounds remain essentially isostructural during the transition. The changes in Fe—N bond length, coordination-sphere distortion and lattice parameters demonstrate the solvate-dependent structural response, whereas Hirshfeld surface, NCI and energy-framework analyses provide a more detailed view of how this response is embedded in the inter­action network. The com­pounds reveal a common feature, *i.e.* a dominant pairwise inter­action along the crystallographic *b* direction. Calculation of this pairwise energy reveals that the tem­per­a­ture dependence of the inter­action energy closely mirrors that of the *b*-axis lattice parameter, whereas no com­parable correlation is observed for the remaining unit-cell parameters. Yet the role of solvent disorder and solvate–solvate inter­actions is unclear. Also, relying solely on crystallographic data makes it difficult to justify statements about hysteresis and incom­plete transitions, and they would need to be fully analysed to understand the nature of the SCO.

## Related literature

The following references are cited in the supporting information: Kratzert *et al.* (2015 ▸); Kratzert & Krossing (2018 ▸).

## Supplementary Material

Crystal structure: contains datablock(s) global, br_100K, br_120K, br_150K, br_160K, br_175K, br_180K, br_190K, br_230K, br_270K, cl_100K, cl_110K, cl_120K, cl_130K, cl_140K, cl_150K, cl_160K, cl_170K, cl_180K, cl_190K, cl_210K, cl_240K, cl_270K, f_100K, f_120K, f_140K, f_150K, f_155K, f_165K, f_175K, f_180K, f_190K, f_230K, f_270K, I_115K, I_135K, I_145K, I_150K, I_155K, I_160K, I_170K, I_200K, I_230K, py_095K, py_115K, py_135K, py_145K, py_150K, py_155K, py_175K, py_195K, py_215K, py_235K, py_255. DOI: 10.1107/S2053229626010740/op3042sup1.cif

Structure factors: contains datablock(s) br_150K. DOI: 10.1107/S2053229626010740/op3042br_150Ksup2.hkl

Structure factors: contains datablock(s) br_160K. DOI: 10.1107/S2053229626010740/op3042br_160Ksup3.hkl

Structure factors: contains datablock(s) br_175K. DOI: 10.1107/S2053229626010740/op3042br_175Ksup4.hkl

Structure factors: contains datablock(s) br_180K. DOI: 10.1107/S2053229626010740/op3042br_180Ksup5.hkl

Structure factors: contains datablock(s) br_190K. DOI: 10.1107/S2053229626010740/op3042br_190Ksup6.hkl

Structure factors: contains datablock(s) br_230K. DOI: 10.1107/S2053229626010740/op3042br_230Ksup7.hkl

Structure factors: contains datablock(s) br_270K. DOI: 10.1107/S2053229626010740/op3042br_270Ksup8.hkl

Structure factors: contains datablock(s) cl_100K. DOI: 10.1107/S2053229626010740/op3042cl_100Ksup9.hkl

Structure factors: contains datablock(s) cl_110K. DOI: 10.1107/S2053229626010740/op3042cl_110Ksup10.hkl

Structure factors: contains datablock(s) cl_120K. DOI: 10.1107/S2053229626010740/op3042cl_120Ksup11.hkl

Structure factors: contains datablock(s) cl_130K. DOI: 10.1107/S2053229626010740/op3042cl_130Ksup12.hkl

Structure factors: contains datablock(s) cl_140K. DOI: 10.1107/S2053229626010740/op3042cl_140Ksup13.hkl

Structure factors: contains datablock(s) cl_150K. DOI: 10.1107/S2053229626010740/op3042cl_150Ksup14.hkl

Structure factors: contains datablock(s) cl_160K. DOI: 10.1107/S2053229626010740/op3042cl_160Ksup15.hkl

Structure factors: contains datablock(s) cl_170K. DOI: 10.1107/S2053229626010740/op3042cl_170Ksup16.hkl

Structure factors: contains datablock(s) cl_180K. DOI: 10.1107/S2053229626010740/op3042cl_180Ksup17.hkl

Structure factors: contains datablock(s) cl_190K. DOI: 10.1107/S2053229626010740/op3042cl_190Ksup18.hkl

Structure factors: contains datablock(s) cl_210K. DOI: 10.1107/S2053229626010740/op3042cl_210Ksup19.hkl

Structure factors: contains datablock(s) cl_240K. DOI: 10.1107/S2053229626010740/op3042cl_240Ksup20.hkl

Structure factors: contains datablock(s) cl_270K. DOI: 10.1107/S2053229626010740/op3042cl_270Ksup21.hkl

Structure factors: contains datablock(s) f_100K. DOI: 10.1107/S2053229626010740/op3042f_100Ksup22.hkl

Structure factors: contains datablock(s) f_120K. DOI: 10.1107/S2053229626010740/op3042f_120Ksup23.hkl

Structure factors: contains datablock(s) f_140K. DOI: 10.1107/S2053229626010740/op3042f_140Ksup24.hkl

Structure factors: contains datablock(s) f_150K. DOI: 10.1107/S2053229626010740/op3042f_150Ksup25.hkl

Structure factors: contains datablock(s) f_155K. DOI: 10.1107/S2053229626010740/op3042f_155Ksup26.hkl

Structure factors: contains datablock(s) f_165K. DOI: 10.1107/S2053229626010740/op3042f_165Ksup27.hkl

Structure factors: contains datablock(s) f_175K. DOI: 10.1107/S2053229626010740/op3042f_175Ksup28.hkl

Structure factors: contains datablock(s) f_180K. DOI: 10.1107/S2053229626010740/op3042f_180Ksup29.hkl

Structure factors: contains datablock(s) f_190K. DOI: 10.1107/S2053229626010740/op3042f_190Ksup30.hkl

Structure factors: contains datablock(s) f_230K. DOI: 10.1107/S2053229626010740/op3042f_230Ksup31.hkl

Structure factors: contains datablock(s) f_270K. DOI: 10.1107/S2053229626010740/op3042f_270Ksup32.hkl

Structure factors: contains datablock(s) I_115K. DOI: 10.1107/S2053229626010740/op3042I_115Ksup33.hkl

Structure factors: contains datablock(s) I_135K. DOI: 10.1107/S2053229626010740/op3042I_135Ksup34.hkl

Structure factors: contains datablock(s) I_145K. DOI: 10.1107/S2053229626010740/op3042I_145Ksup35.hkl

Structure factors: contains datablock(s) I_150K. DOI: 10.1107/S2053229626010740/op3042I_150Ksup36.hkl

Structure factors: contains datablock(s) I_155K. DOI: 10.1107/S2053229626010740/op3042I_155Ksup37.hkl

Structure factors: contains datablock(s) I_160K. DOI: 10.1107/S2053229626010740/op3042I_160Ksup38.hkl

Structure factors: contains datablock(s) I_170K. DOI: 10.1107/S2053229626010740/op3042I_170Ksup39.hkl

Structure factors: contains datablock(s) I_200K. DOI: 10.1107/S2053229626010740/op3042I_200Ksup40.hkl

Structure factors: contains datablock(s) I_230K. DOI: 10.1107/S2053229626010740/op3042I_230Ksup41.hkl

Structure factors: contains datablock(s) py_095K. DOI: 10.1107/S2053229626010740/op3042py_095Ksup42.hkl

Structure factors: contains datablock(s) py_115K. DOI: 10.1107/S2053229626010740/op3042py_115Ksup43.hkl

Structure factors: contains datablock(s) py_135K. DOI: 10.1107/S2053229626010740/op3042py_135Ksup44.hkl

Structure factors: contains datablock(s) py_145K. DOI: 10.1107/S2053229626010740/op3042py_145Ksup45.hkl

Structure factors: contains datablock(s) py_150K. DOI: 10.1107/S2053229626010740/op3042py_150Ksup46.hkl

Structure factors: contains datablock(s) py_155K. DOI: 10.1107/S2053229626010740/op3042py_155Ksup47.hkl

Structure factors: contains datablock(s) py_175K. DOI: 10.1107/S2053229626010740/op3042py_175Ksup48.hkl

Structure factors: contains datablock(s) py_195K. DOI: 10.1107/S2053229626010740/op3042py_195Ksup49.hkl

Structure factors: contains datablock(s) py_215K. DOI: 10.1107/S2053229626010740/op3042py_215Ksup50.hkl

Structure factors: contains datablock(s) py_235K. DOI: 10.1107/S2053229626010740/op3042py_235Ksup51.hkl

Structure factors: contains datablock(s) py_255. DOI: 10.1107/S2053229626010740/op3042py_255sup52.hkl

Structure factors: contains datablock(s) br_100K. DOI: 10.1107/S2053229626010740/op3042br_100Ksup53.hkl

Structure factors: contains datablock(s) br_120K. DOI: 10.1107/S2053229626010740/op3042br_120Ksup54.hkl

Supporting information file. DOI: 10.1107/S2053229626010740/op3042sup55.pdf

CCDC references: 2565651, 2565652, 2565653, 2565654, 2565655, 2565656, 2565657, 2565658, 2565659, 2565660, 2565661, 2565637, 2565638, 2565639, 2565640, 2565641, 2565642, 2565643, 2565644, 2565645, 2565646, 2565647, 2565648, 2565649, 2565626, 2565627, 2565628, 2565629, 2565630, 2565631, 2565632, 2565633, 2565634, 2570240, 2570241, 2570242, 2570243, 2570244, 2570245, 2570246, 2570247, 2570248, 2565664, 2565665, 2565666, 2565667, 2565668, 2565669, 2565670, 2565671, 2565672, 2565673, 2565674

## Figures and Tables

**Figure 1:**
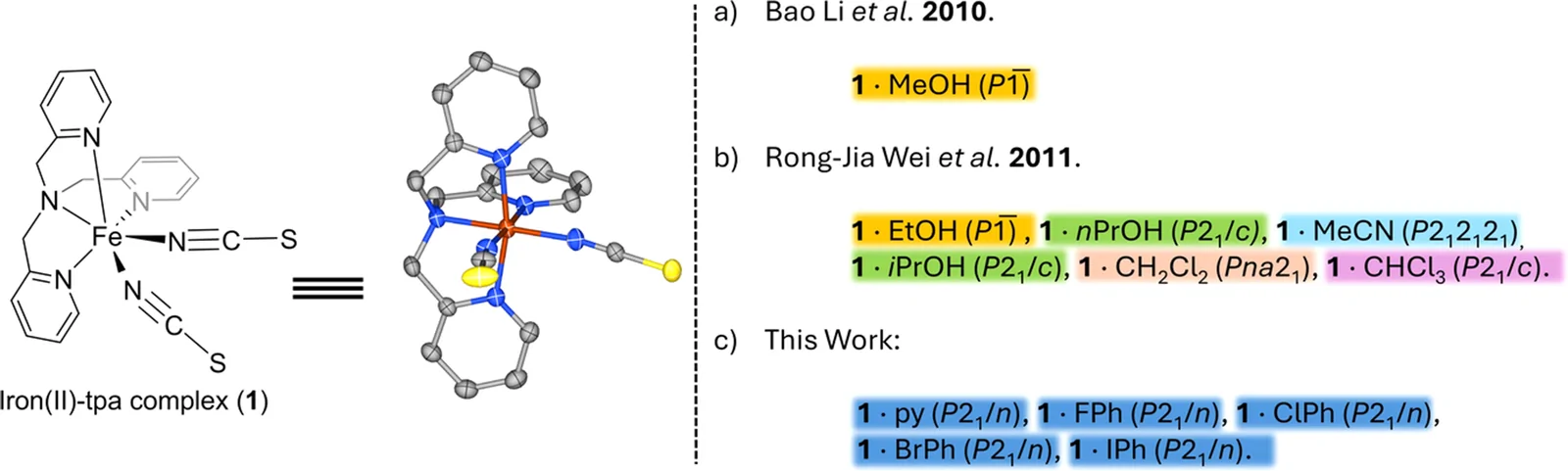
(Left) The mol­ecular structure and representative ellipsoid plot (50% probability level) of [Fe(NCS)_2_(tpa)]. (Right) Overview of the reported sol­vato­morphs of com­pound **1**, with the isostructural com­pounds colour-coded.

**Figure 2:**
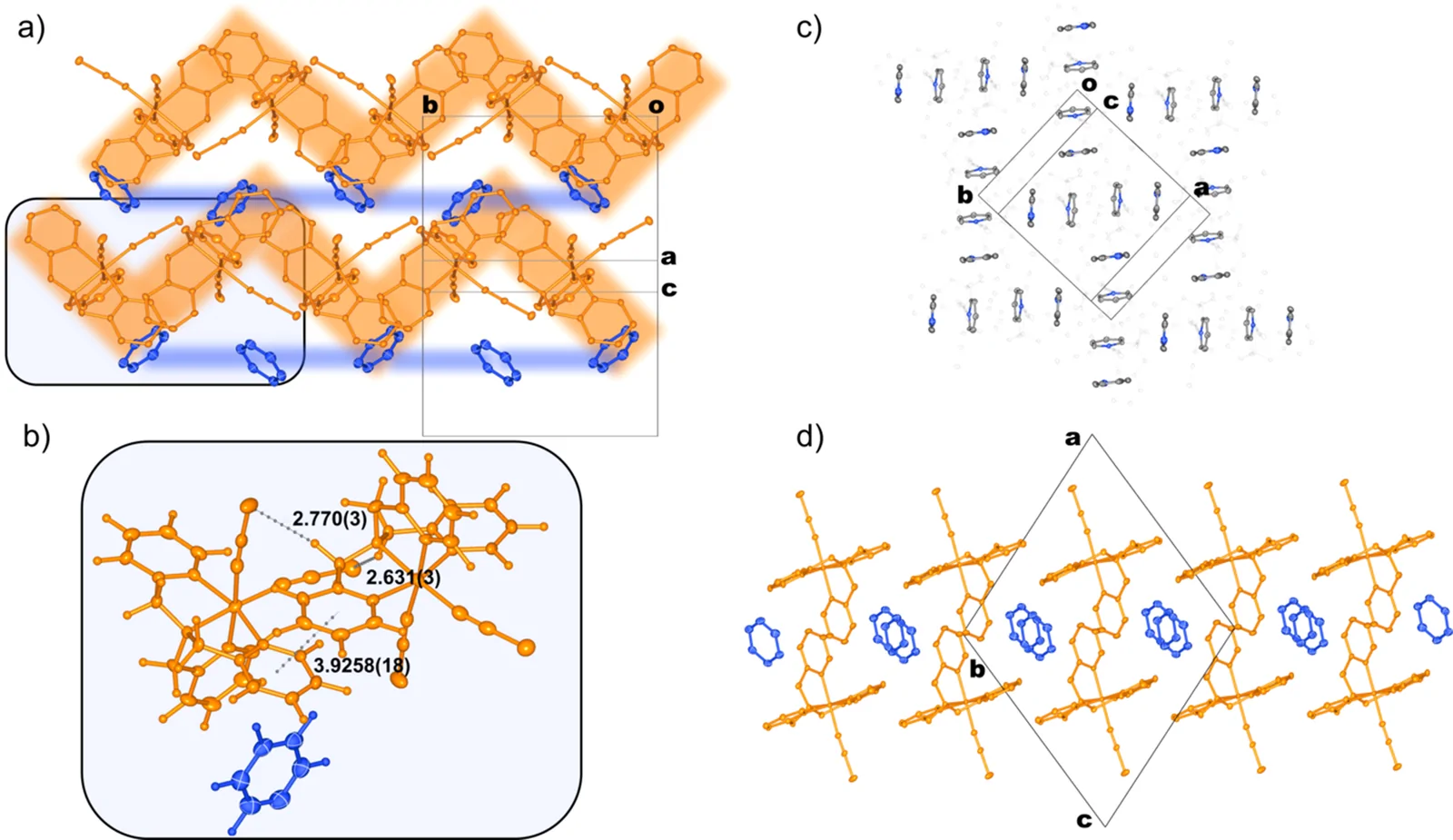
Packing diagrams of **1**·py at 95 K, with displacement ellipsoids drawn at the 50% probability level. (*a*) View along the *ac* diagonal, with the zigzag chain of the com­plex unit highlighted in orange and the direction in which the pyridine units propagate highlighted in blue. (*b*) Zoomed view of the arrangement of two neighbouring com­plexes, with close contacts annotated. (*c*) View approximately along *c*, highlighting the π-stacking between the solvate and the com­plexes. (*d*) View along the *b* axis. Only **1**·py is shown, but the description applies to all isostructural com­pounds.

**Figure 3:**
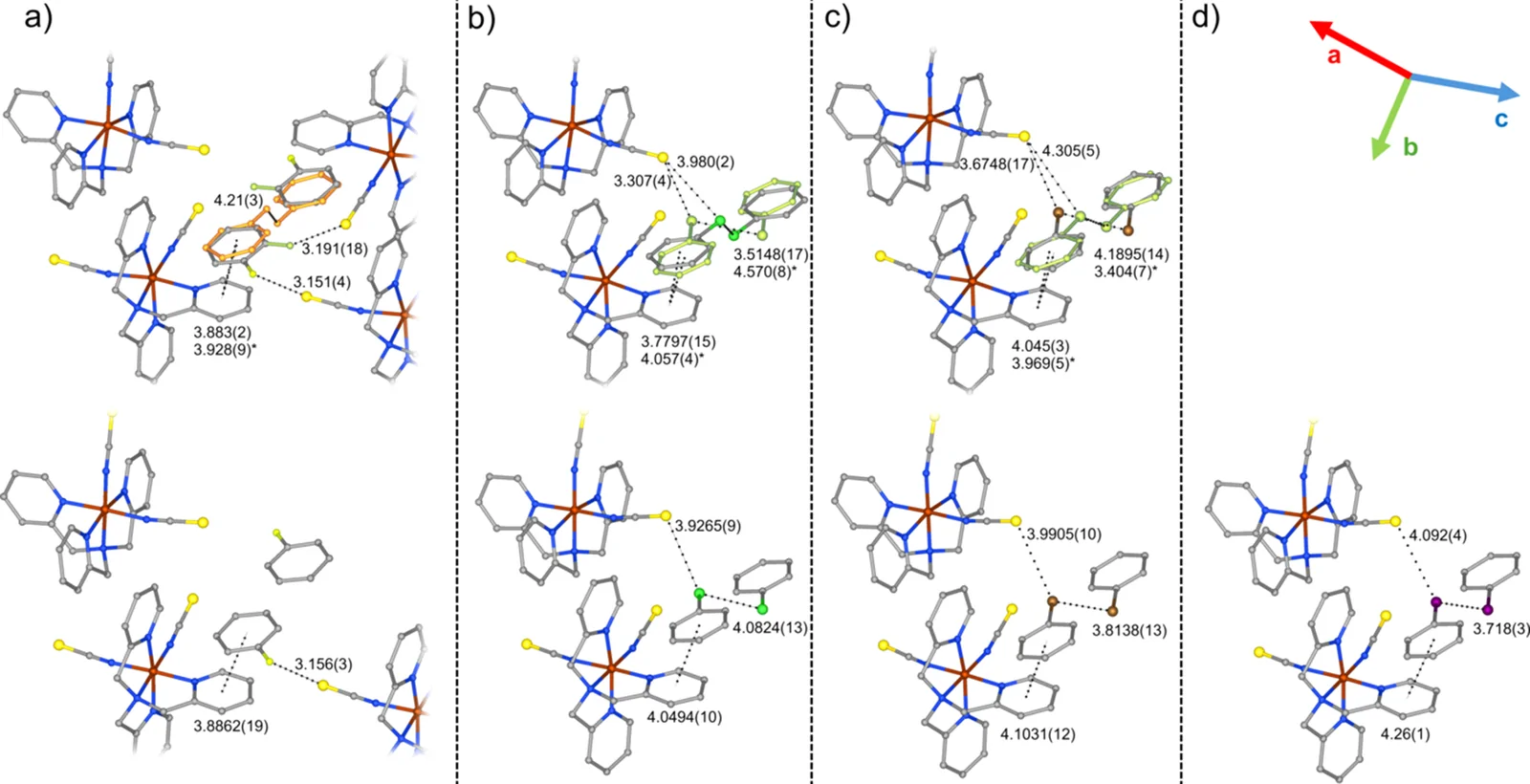
The mol­ecular arrangements of the halide-containing solvates, before the transition (upper row) and after the transition (lower row). Structures are drawn in ball-and-stick style and H atoms have been omitted. The first minor part is drawn in green and the second minor part in orange (only for **1**·FPh). For parts (*a*) to (*d*): **1**·FPh at 155 and 150 K, **1**·ClPh at 170 and 160 K, **1**·BrPh at 190 and 180 K, and **1**·IPh at 150 K only.

**Figure 4:**
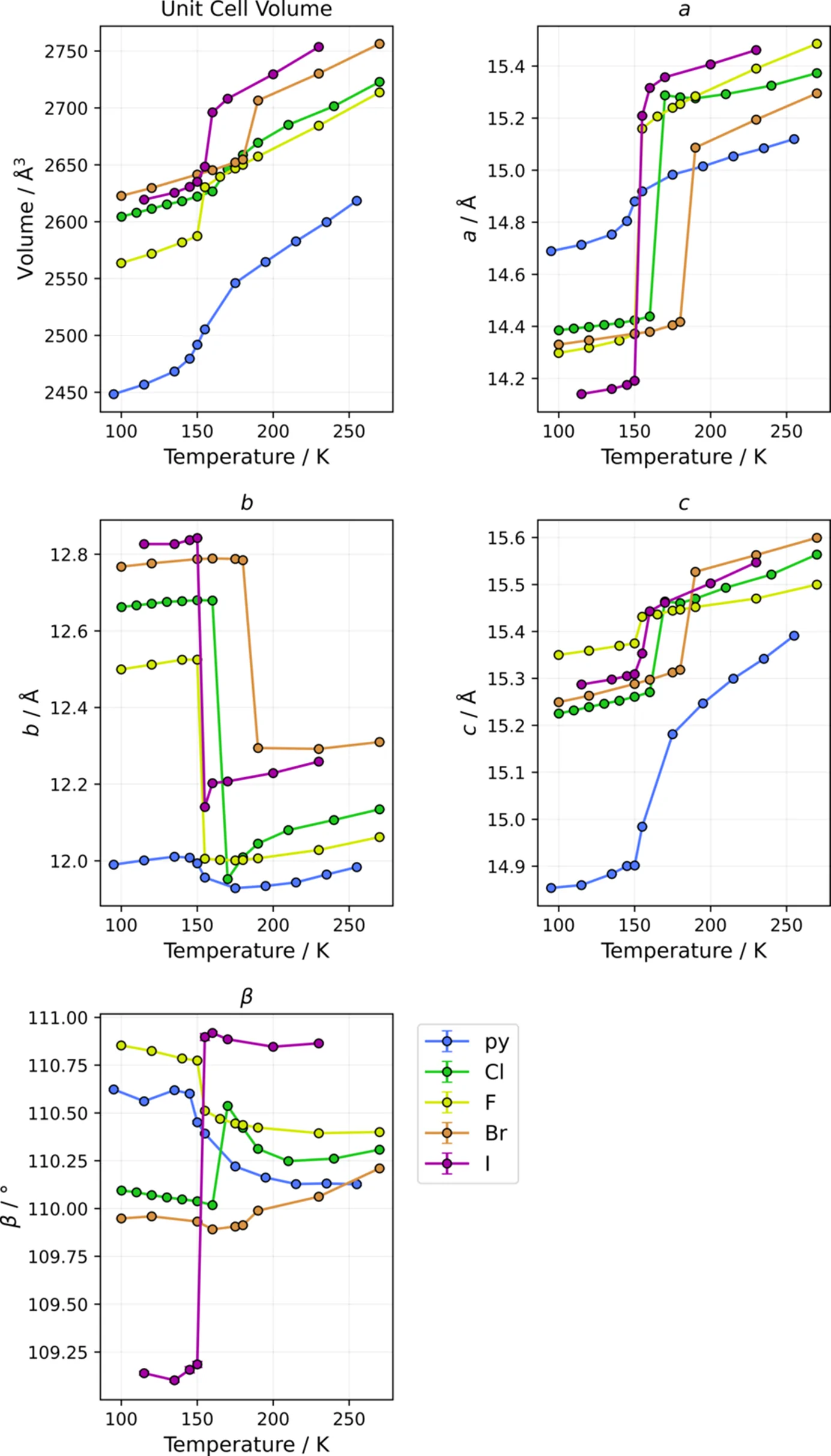
Lattice parameters as a function of the tem­per­a­ture. ESD-based uncertainty bars are shown but are obstructed by the marker size in many cases.

**Figure 5:**
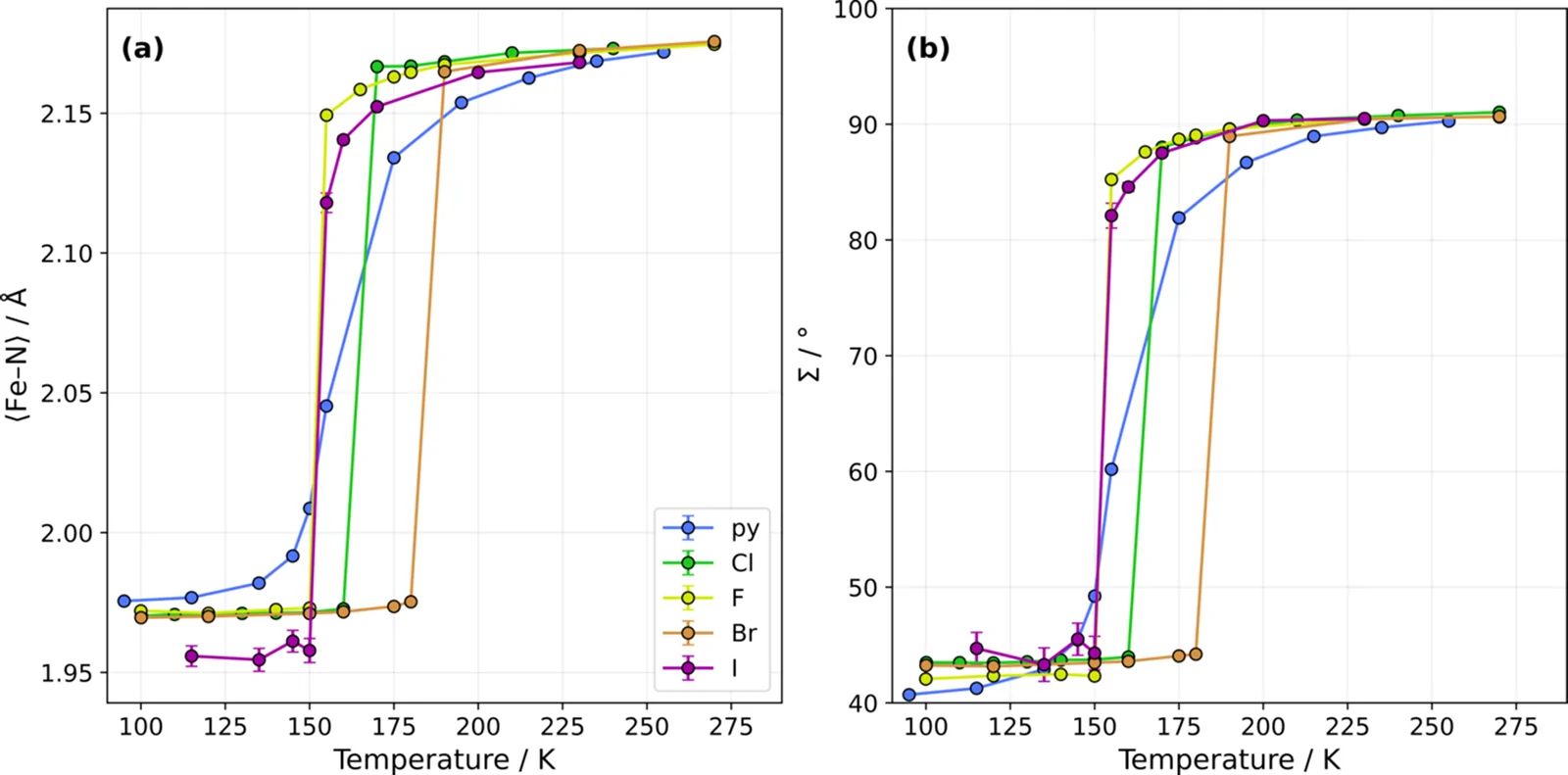
(*a*) Temperature evolution of the average Fe—N distances. (*b*) Temperature dependence of the octa­hedral distortion. Errors on the values were propagated.

**Figure 6:**
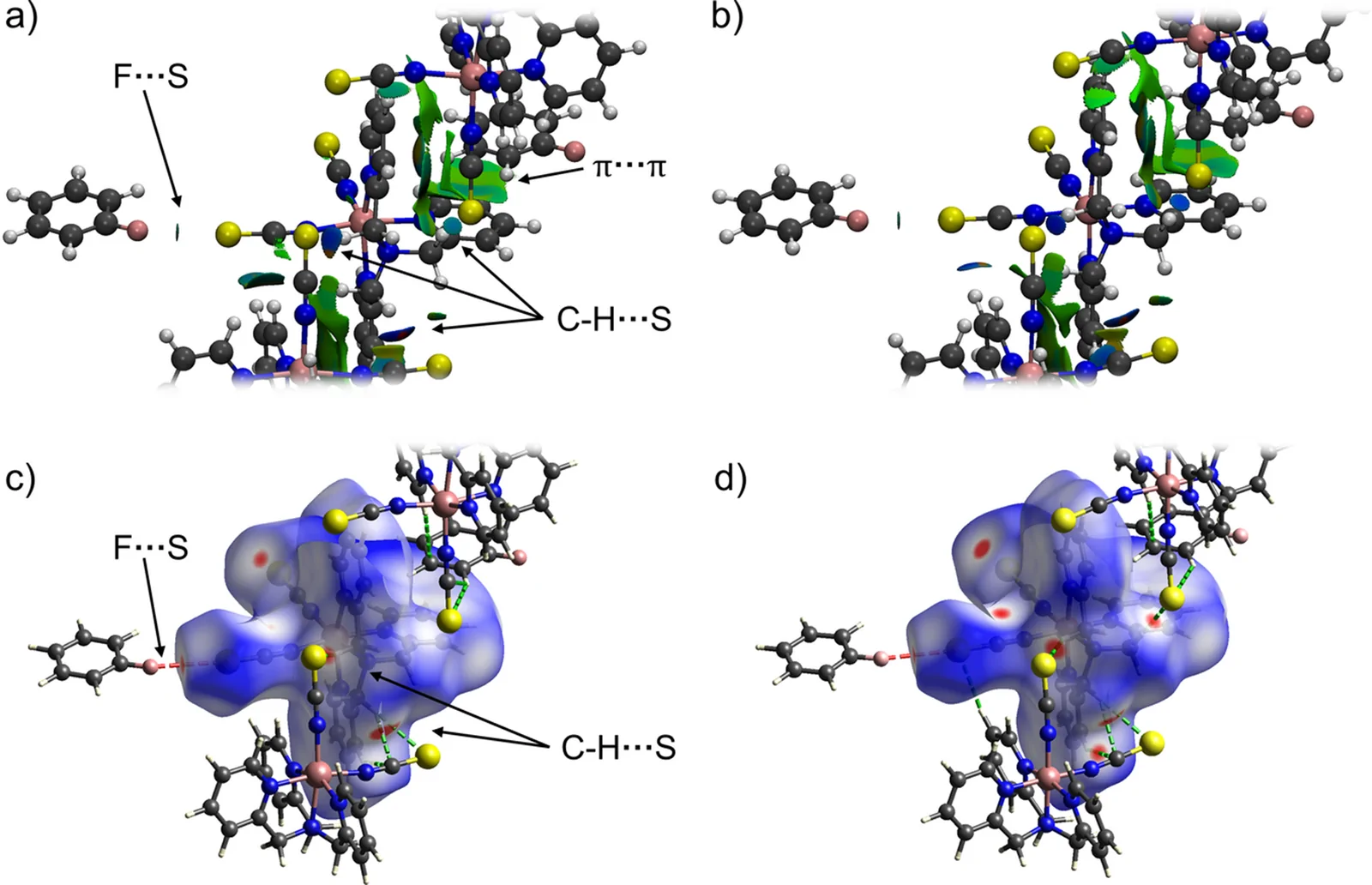
NCI plots of **1**·FPh, highlighting the relevant short contacts at (*a*) 155 and (*b*) 150 K, the RDG is cut at 0.4, while sign(λ)ρ is colour-coded from −0.015 (blue) *via* 0 (green) to 0.015 a.u. (red). Views of the Hirshfeld surface mapped with *d*_norm_ at (*c*) 155 and (*d*) 150 K.

**Figure 7:**
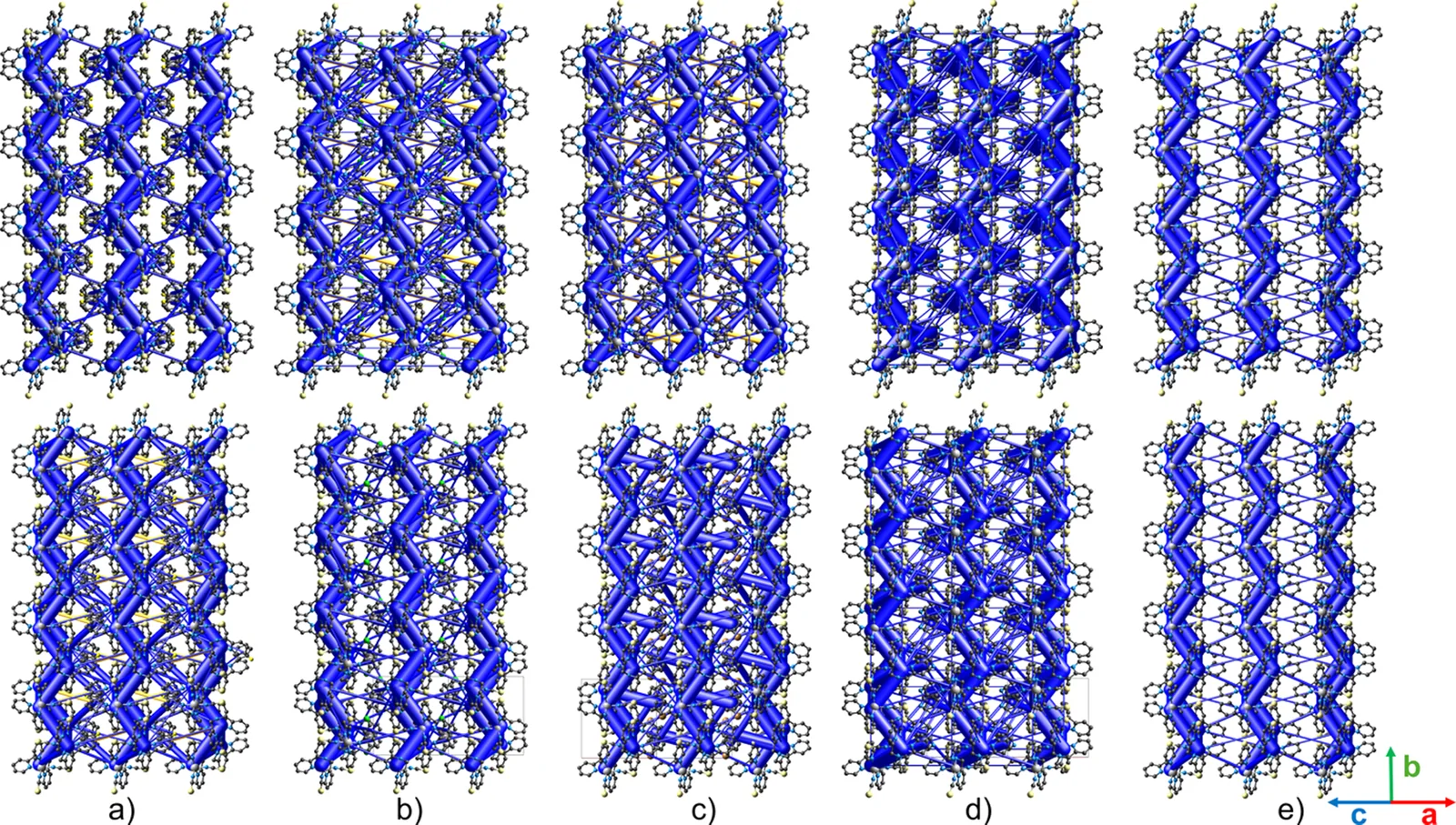
Frameworks of the total energy, with a tube size of 80 and a low-energy cut-off of 10 kJ mol^−1^. Parts (*a*) to (*e*) show the F, Cl, Br, py and I variants, respectively; the upper row is before and the lower row is after the transition. H atoms have been omitted for clarity.

**Figure 8:**
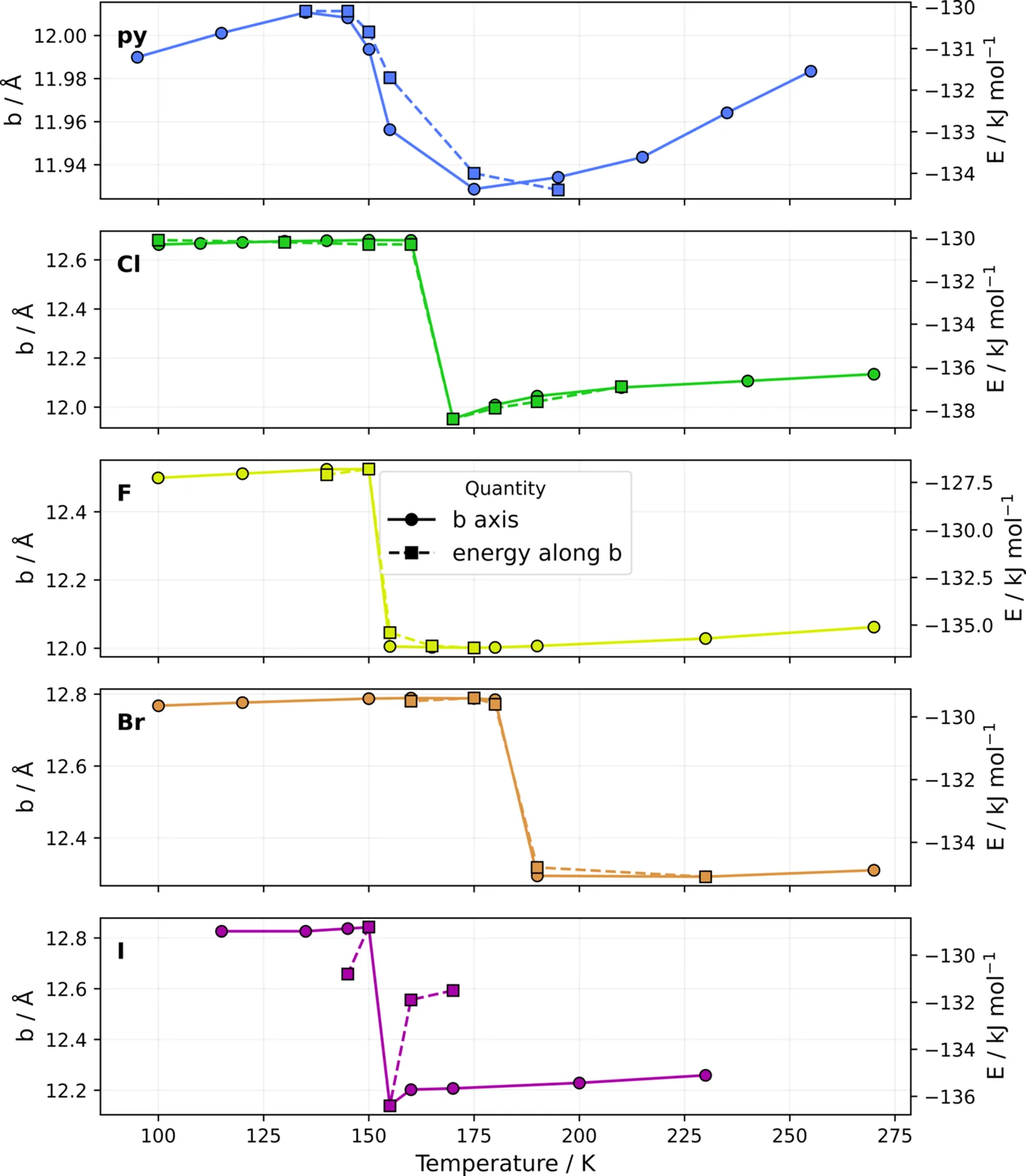
The tem­per­a­ture dependence of the *b* unit-cell parameter (circles), together with the pairwise inter­action energy of **1** (squares) with its next neighbour generated by the symmetry operation (−*x* + 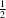, *y* + 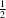, −*z* + 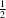). Errors on *b* are shown but hidden behind the marker.

## Data Availability

The supplementary material contains additional crystallographic information and data that support the findings of this study.
