# Supplementary material for: Analysis of lattice changes and weak inter­actions in a series of sol­vato­morphic isostructural spin-crossover com­pounds
Source: Acta Crystallogr C Struct Chem. 2026 Oct 20;82(Pt 11):723–33. doi: 10.1107/S2053229626010740 (PMC13735075; doi:10.1107/S2053229626010740)
Supplement: Supplementary file 1 [file c-82-00723-sup55.pdf]

Supporting Information: Analysis of Lattice Changes and Weak Interactions in a Series of Solvatomorphic Isostructural Spin-Crossover Compounds

Daniel Brüx, Johannes Bartusel and Prof. Dr. Florian Kleemiss*

Institute of Inorganic Chemistry, RWTH Aachen University, Landoltweg 1a, 52074 Aachen, Germany
*Corresponding author: florian.kleemiss@ac.rwth-aachen.de

1 Representative Diffraction Pattern

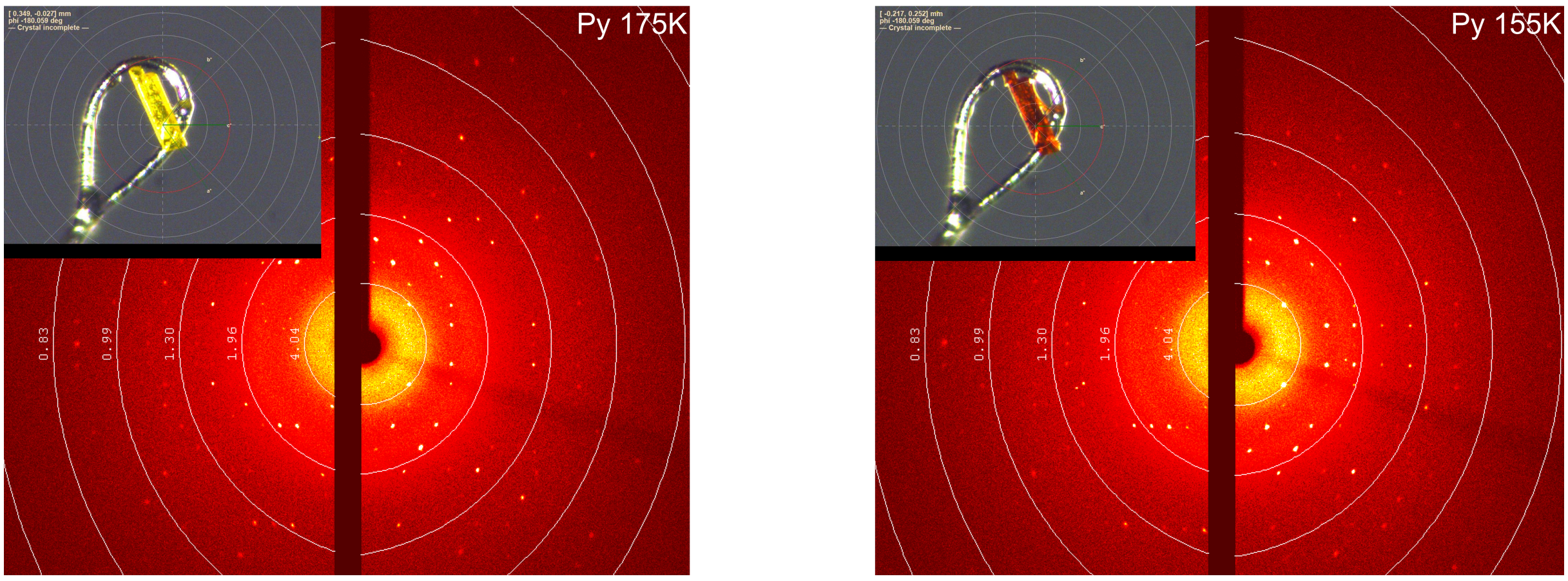

Fig. S1: Reflection pattern and crystal image of **1** · py before (left) and after (right) the transition.

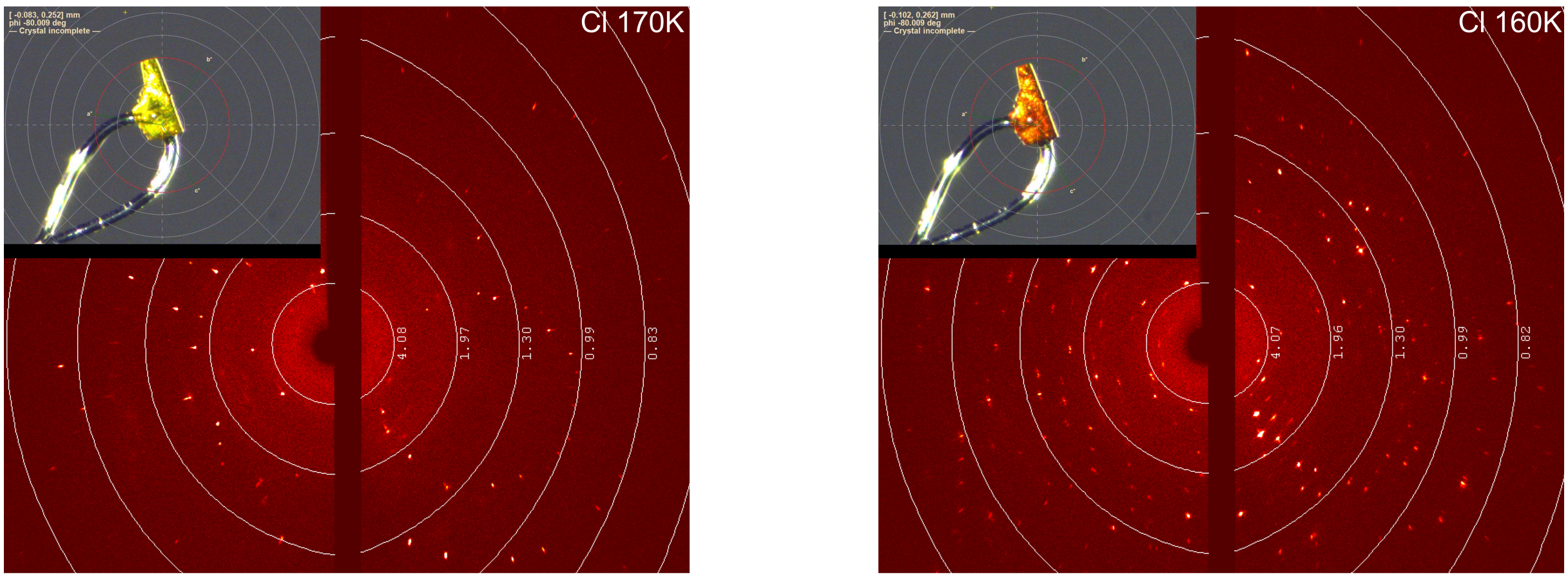

Fig. S2: Reflection pattern and crystal image of **1** · ClPh before (left) and after (right) the transition.

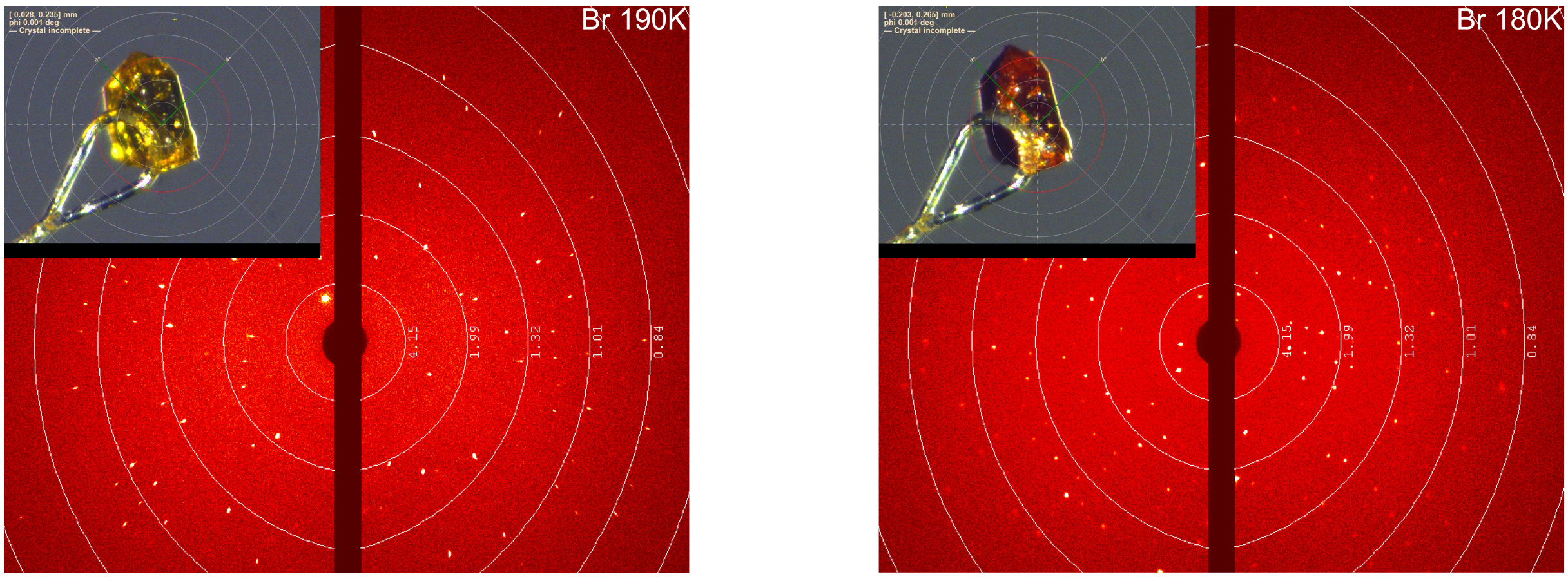

Fig. S3: Reflection pattern and crystal image of **1** · BrPh before (left) and after (right) the transition.

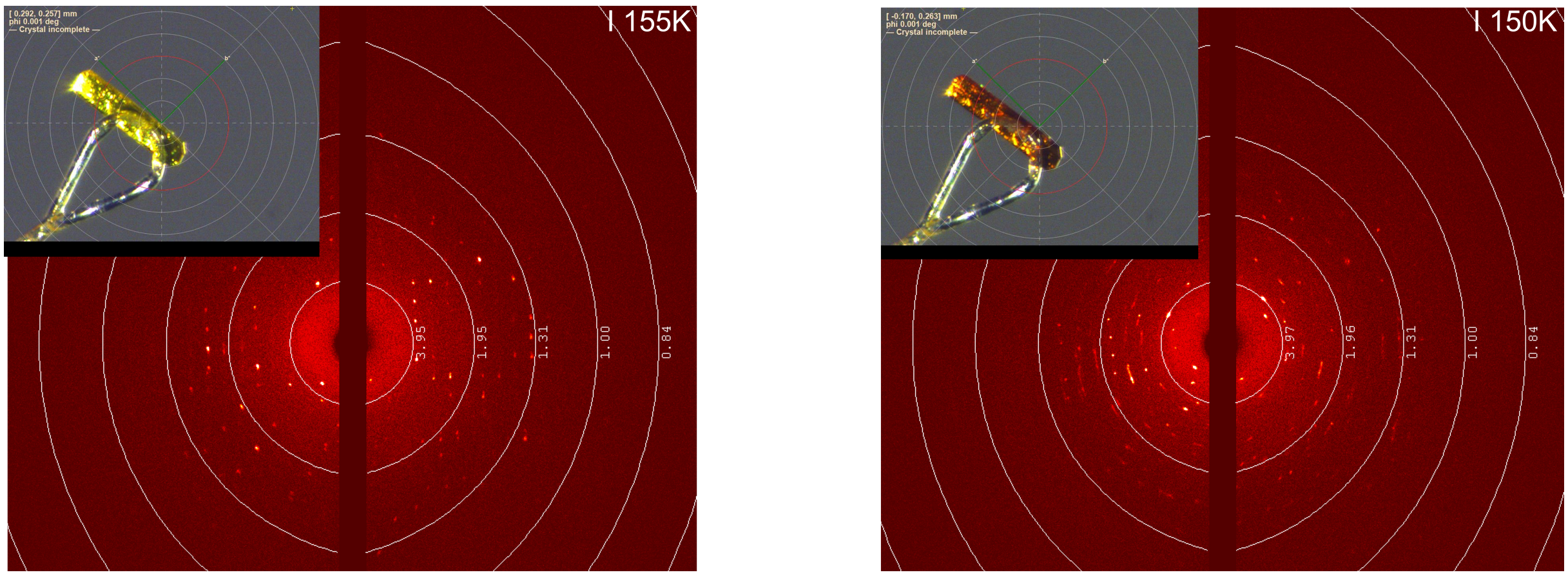

Fig. S4: Reflection pattern and crystal image of **1** · IPh before (left) and after (right) the transition.

1.1 Powder Diffracition of 1 · py

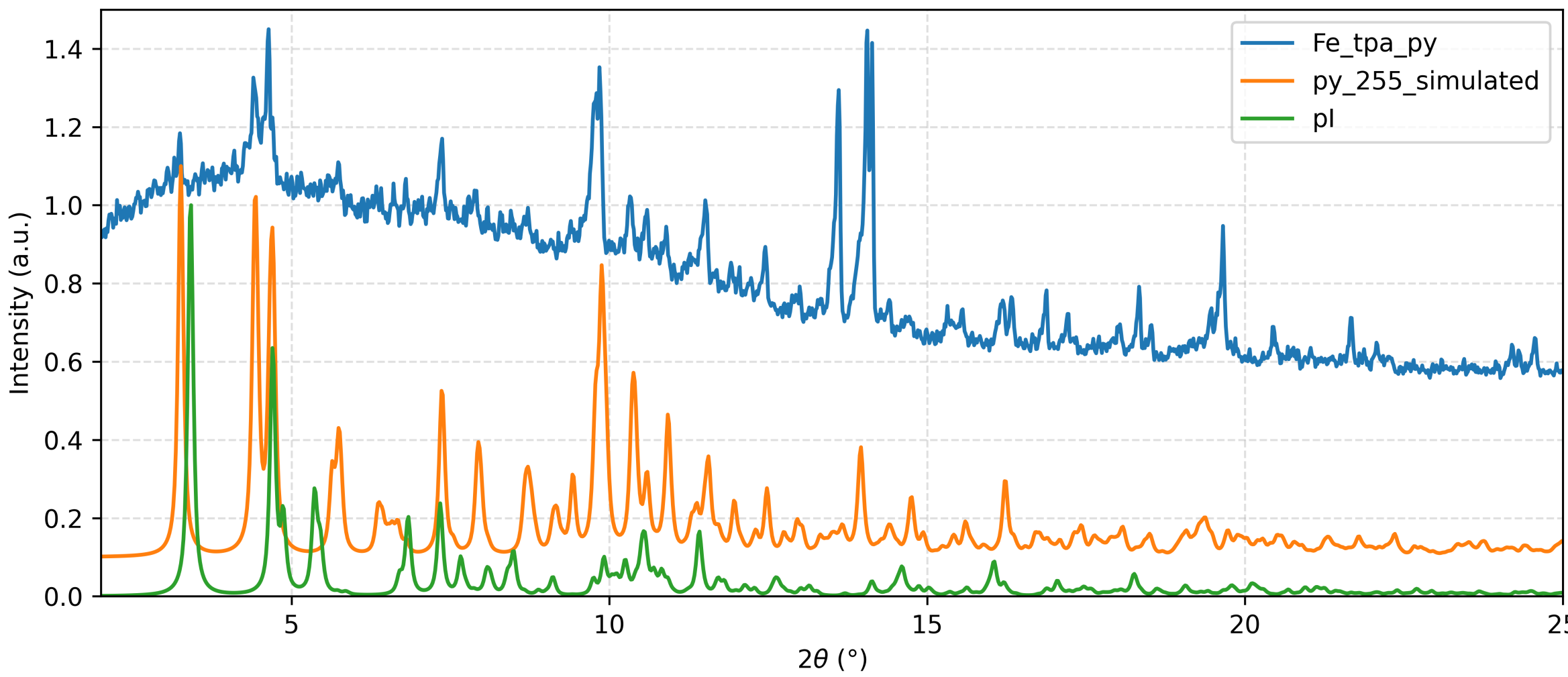

Fig. S5: Room temperature powder diffraction pattern of **1** · py; Simulated patterns from our SCXRD study and pI from Li *et al.*[1] are displayed for comparison. Data were recorded on a flat sample with a STOE STADI MP diffractometer with an imageplate detector using Mo-Kα radiation exposing for 2 h.

2 Crystallographic Information

Table S 1: Crystallographic data and structure-refinement parameters for **1** · FPh from 100 to 155 K.

CCDC ref. code	2565651	2565652	2565653	2565654	2565655
Temperature / K	100	120	140	150	155
Space group	$P2_1/n$	$P2_1/n$	$P2_1/n$	$P2_1/n$	$P2_1/n$
a / Å	14.2977(3)	14.3180(3)	14.3456(3)	14.3703(3)	15.1594(3)
b / Å	12.4994(3)	12.5119(3)	12.5246(3)	12.5251(3)	12.0053(2)
c / Å	15.3500(3)	15.3591(3)	15.3694(3)	15.3745(4)	15.4313(3)
β / °	110.853(2)	110.824(2)	110.785(2)	110.774(3)	110.511(2)
V / Å^3	2563.55(10)	2571.77(10)	2581.74(10)	2587.34(12)	2630.35(9)
ρ_{calc} / g cm^{-3}	1.447	1.442	1.437	1.434	1.410
μ / mm^{-1}	0.785	0.783	0.780	0.778	0.765
$F(000)$	1155.072	1155.072	1155.072	1155.072	1155.072
θ range / °	2.16–28.28	2.16–28.28	2.16–28.28	2.16–28.28	2.21–28.28
Reflections collected	71141	72319	72682	74232	76654
Independent reflections	6360	6382	6413	6434	6525
R_{int}	0.0291	0.0289	0.0291	0.0300	0.0134
Data/restraints/parameters	6360/0/325	6382/0/325	6413/0/325	6434/0/325	6525/149/408
Goodness-of-fit on F^2	1.0305	0.9613	1.0419	1.0372	1.0452
R_1 $[I > 2\sigma(I)]$	0.0476	0.0480	0.0487	0.0507	0.0404
wR_2 $[I > 2\sigma(I)]$	0.1200	0.1280	0.1250	0.1302	0.1018
R_1 (all data)	0.0508	0.0512	0.0526	0.0551	0.0425
wR_2 (all data)	0.1220	0.1304	0.1275	0.1331	0.1031

Table S 2: Crystallographic data and structure-refinement parameters for **1** · FPh from 165 to 230 K.

CCDC ref. code	2565656	2565657	2565658	2565659	2565660
Temperature / K	165	175	180	190	230
Space group	$P2_1/n$	$P2_1/n$	$P2_1/n$	$P2_1/n$	$P2_1/n$
a / Å	15.2066(3)	15.2396(3)	15.2539(3)	15.2839(3)	15.3904(3)
b / Å	12.0024(2)	12.0011(2)	12.0022(2)	12.0065(2)	12.0283(2)
c / Å	15.4362(3)	15.4444(3)	15.4464(3)	15.4519(3)	15.4700(3)
β / °	110.469(2)	110.445(2)	110.437(2)	110.423(2)	110.394(2)
V / Å^3	2639.47(9)	2646.73(9)	2649.93(9)	2657.28(9)	2684.30(9)
ρ_{calc} / g cm^{-3}	1.405	1.402	1.400	1.396	1.382
μ / mm^{-1}	0.763	0.761	0.760	0.758	0.750
$F(000)$	1155.072	1155.072	1155.072	1155.072	1155.072
θ range / °	2.21–28.28	2.20–28.28	2.20–28.28	2.20–28.28	2.20–28.28
Reflections collected	76127	76659	76682	97972	77392
Independent reflections	6545	6558	6565	6584	6649
R_{int}	0.0134	0.0130	0.0130	0.0203	0.0134
Data/restraints/parameters	6545/155/408	6558/149/408	6565/149/408	6584/149/408	6649/149/408
Goodness-of-fit on F^2	1.0446	1.0506	1.0502	1.0455	1.0508
R_1 $[I > 2\sigma(I)]$	0.0350	0.0323	0.0317	0.0303	0.0332
wR_2 $[I > 2\sigma(I)]$	0.0928	0.0889	0.0885	0.0883	0.0964
R_1 (all data)	0.0370	0.0349	0.0341	0.0327	0.0368
wR_2 (all data)	0.0940	0.0906	0.0901	0.0898	0.0990

Table S 3: Crystallographic data and structure-refinement parameters for **1** · FPh at 270 K.

CCDC ref. code	2565661
Temperature / K	270
Space group	$P2_1/n$
a / Å	15.4857(3)
b / Å	12.0619(2)
c / Å	15.4996(4)
β / °	110.400(2)
V / Å^3	2713.55(10)
ρ_{calc} / g cm^{-3}	1.367
μ / mm^{-1}	0.742
$F(000)$	1155.072
θ range / °	2.19–28.28
Reflections collected	78108
Independent reflections	6740
R_{int}	0.0140
Data/restraints/parameters	6740/149/408
Goodness-of-fit on F^2	1.0437
R_1 [$I > 2\sigma(I)$]	0.0366
wR_2 [$I > 2\sigma(I)$]	0.1096
R_1 (all data)	0.0415
wR_2 (all data)	0.1132

Table S 4: Crystallographic data and structure-refinement parameters for **1** · ClPh from 100 to 145 K.

CCDC ref. code	2565637	2565638 K	2565639 K	2565640 K	2565641
Temperature / K	100	110	120	130	140
Space group	$P2_1/n$	$P2_1/n$	$P2_1/n$	$P2_1/n$	$P2_1/n$
a / Å	14.3851(2)	14.3918(3)	14.3978(3)	14.4057(3)	14.4125(3)
b / Å	12.6621(2)	12.6666(2)	12.6710(2)	12.6757(2)	12.6776(2)
c / Å	15.2251(3)	15.2318(3)	15.2389(3)	15.2461(3)	15.2525(3)
β / °	110.094(2)	110.085(2)	110.070(2)	110.058(2)	110.048(2)
V / Å^3	2604.38(8)	2607.82(9)	2611.28(9)	2615.11(9)	2618.01(9)
ρ_{calc} / g cm^{-3}	1.466	1.464	1.462	1.460	1.459
μ / mm^{-1}	0.869	0.868	0.867	0.866	0.865
$F(000)$	1187.598	1187.598	1187.598	1187.598	1187.598
θ range / °	2.15–28.28	2.15–28.28	2.15–28.28	2.15–28.28	2.14–28.28
Reflections collected	76006	75361	76156	76285	76522
Independent reflections	6471	6480	6487	6493	6505
R_{int}	0.0252	0.0250	0.0250	0.0251	0.0247
Data/restraints/parameters	6471/0/325	6480/0/325	6487/0/325	6493/0/325	6505/0/325
Goodness-of-fit on F^2	1.0263	1.0298	1.0255	1.0432	1.0279
R_1 [$I > 2\sigma(I)$]	0.0243	0.0257	0.0267	0.0283	0.0295
wR_2 [$I > 2\sigma(I)$]	0.0632	0.0668	0.0700	0.0744	0.0776
R_1 (all data)	0.0277	0.0293	0.0304	0.0321	0.0337
wR_2 (all data)	0.0653	0.0693	0.0724	0.0770	0.0807

Table S 5: Crystallographic data and structure-refinement parameters for **1** · ClPh from 150 to 190 K.

CCDC ref. code	2565642	2565643 K	2565644 K	2565645	2565646
Temperature / K	150	160	170	180	190
Space group	$P2_1/n$	$P2_1/n$	$P2_1/n$	$P2_1/n$	$P2_1/n$
a / Å	14.4232(3)	14.4380(3)	15.2878(2)	15.2799(3)	15.2759(2)
b / Å	12.6801(2)	12.6792(2)	11.9522(1)	12.0091(2)	12.0449(1)
c / Å	15.2608(3)	15.2705(3)	15.4638(2)	15.4602(2)	15.4697(2)
β / °	110.038(2)	110.018(2)	110.537(2)	110.422(2)	110.312(2)
V / Å^3	2622.06(9)	2626.57(9)	2646.01(6)	2658.61(8)	2669.38(6)
ρ_{calc} / g cm^{-3}	1.456	1.454	1.443	1.436	1.431
μ / mm^{-1}	0.863	0.862	0.855	0.851	0.848
$F(000)$	1187.598	1187.598	1187.598	1187.598	1187.598
θ range / °	2.14–28.28	2.14–28.28	2.21–28.28	2.20–28.28	2.20–28.28
Reflections collected	76025	76542	78174	79036	78961
Independent reflections	6515	6526	6554	6587	6617
R_{int}	0.0249	0.0252	0.0134	0.0130	0.0119
Data/restraints/parameters	6515/0/325	6526/0/325	6554/39/387	6587/4/375	6617/2/375
Goodness-of-fit on F^2	1.0280	1.0386	1.0418	1.0494	1.0395
R_1 [$I > 2\sigma(I)$]	0.0305	0.0331	0.0303	0.0278	0.0265
wR_2 [$I > 2\sigma(I)$]	0.0808	0.0880	0.0746	0.0673	0.0629
R_1 (all data)	0.0351	0.0379	0.0322	0.0298	0.0284
wR_2 (all data)	0.0843	0.0915	0.0758	0.0684	0.0640

Table S 6: Crystallographic data and structure-refinement parameters for **1** · ClPh from 210 to 270 K.

Parameter	2565647 K	2565648	2565649
Temperature / K	210	240	270
Space group	$P2_1/n$	$P2_1/n$	$P2_1/n$
a / Å	15.2919(2)	15.3247(3)	15.3728(3)
b / Å	12.0800(1)	12.1065(2)	12.1343(2)
c / Å	15.4930(2)	15.5211(2)	15.5638(3)
β / °	110.248(2)	110.261(2)	110.308(2)
V / Å^3	2685.10(6)	2701.43(9)	2722.78(9)
ρ_{calc} / g cm^{-3}	1.422	1.414	1.403
μ / mm^{-1}	0.843	0.838	0.831
$F(000)$	1187.598	1187.598	1187.598
θ range / °	2.19–28.28	2.19–28.28	2.18–28.28
Reflections collected	78410	82537	80082
Independent reflections	6660	6713	6761
R_{int}	0.0124	0.0118	0.0121
Data/restraints/parameters	6660/2/375	6713/2/375	6761/9/375
Goodness-of-fit on F^2	1.0536	1.0320	1.0340
R_1 [$I > 2\sigma(I)$]	0.0261	0.0286	0.0335
wR_2 [$I > 2\sigma(I)$]	0.0651	0.0739	0.0940
R_1 (all data)	0.0283	0.0311	0.0369
wR_2 (all data)	0.0664	0.0754	0.0967

Table S 7: Crystallographic data and structure-refinement parameters for **1** · BrPh from 100 to 175 K.

CCDC ref. code	2565626	2565627	2565628	2565629	2565630
Temperature / K	100	120	150	160	175
Space group	$P2_1/n$	$P2_1/n$	$P2_1/n$	$P2_1/n$	$P2_1/n$
a / Å	14.3302(3)	14.3465(3)	14.3721(4)	14.3790(4)	14.4045(4)
b / Å	12.7675(2)	12.7762(2)	12.7874(3)	12.7888(3)	12.7878(3)
c / Å	15.2494(4)	15.2630(4)	15.2882(4)	15.2972(4)	15.3126(4)
β / °	109.948(3)	109.959(3)	109.932(3)	109.891(3)	109.906(3)
V / Å^3	2622.65(11)	2629.58(11)	2641.39(13)	2645.19(13)	2652.09(13)
ρ_{calc} / g cm^{-3}	1.569	1.565	1.558	1.555	1.551
μ / mm^{-1}	2.290	2.284	2.273	2.270	2.264
$F(000)$	1258.243	1258.243	1258.243	1258.243	1258.243
θ range / °	2.14–28.28	2.13–28.28	2.13–28.28	2.13–28.28	2.13–28.28
Reflections collected	65301	65396	64978	65622	65372
Independent reflections	6503	6520	6551	6560	6581
R_{int}	0.0238	0.0253	0.0268	0.0269	0.0277
Data/restraints/parameters	6503/0/335	6520/0/335	6551/0/335	6560/0/335	6581/0/335
Goodness-of-fit on F^2	1.0475	1.0418	1.0460	1.0495	1.0439
R_1 [$I > 2\sigma(I)$]	0.0221	0.0238	0.0284	0.0290	0.0305
wR_2 [$I > 2\sigma(I)$]	0.0501	0.0548	0.0646	0.0649	0.0694
R_1 (all data)	0.0287	0.0320	0.0386	0.0401	0.0435
wR_2 (all data)	0.0523	0.0580	0.0690	0.0696	0.0751

Table S 8: Crystallographic data and structure-refinement parameters for **1** · BrPh from 180 to 270 K.

CCDC ref. code	2565631	2565632	2565633	2565634
Temperature / K	180	190	230	270
Space group	$P2_1/n$	$P2_1/n$	$P2_1/n$	$P2_1/n$
a / Å	14.4172(4)	15.0867(4)	15.1940(4)	15.2951(5)
b / Å	12.7845(3)	12.2943(2)	12.2919(3)	12.3099(3)
c / Å	15.3183(4)	15.5270(3)	15.5626(4)	15.5994(5)
β / °	109.913(3)	109.989(3)	110.062(3)	110.210(4)
V / Å^3	2654.61(13)	2706.46(11)	2730.16(13)	2756.25(16)
ρ_{calc} / g cm^{-3}	1.550	1.520	1.507	1.493
μ / mm^{-1}	2.262	2.219	2.199	2.179
$F(000)$	1258.243	1258.243	1258.243	1258.243
θ range / °	2.13–28.28	2.17–28.28	2.16–28.28	2.16–28.28
Reflections collected	66099	66000	68518	68934
Independent reflections	6586	6728	6781	6846
R_{int}	0.0280	0.0173	0.0208	0.0194
Data/restraints/parameters	6586/0/335	6728/30/387	6781/264/358	6846/34/406
Goodness-of-fit on F^2	1.0419	1.0321	1.0317	1.0495
R_1 [$I > 2\sigma(I)$]	0.0320	0.0301	0.0328	0.0294
wR_2 [$I > 2\sigma(I)$]	0.0733	0.0634	0.0763	0.0705
R_1 (all data)	0.0460	0.0365	0.0417	0.0422
wR_2 (all data)	0.0799	0.0661	0.0812	0.0765

Table S 9: Crystallographic data and structure-refinement parameters for **1** · IPh from 115 to 155 K.

Parameter	2570240	2570241	2570242	2570243	2570244
Temperature / K	115	135	145	150	155
Space group	$P2_1/n$	$P2_1/n$	$P2_1/n$	$P2_1/n$	$P2_1/n$
a / Å	14.1401(13)	14.1597(14)	14.175(2)	14.191(2)	15.209(3)
b / Å	12.8265(9)	12.8265(9)	12.8369(13)	12.8422(13)	12.1403(14)
c / Å	15.2870(13)	15.2977(13)	15.305(2)	15.309(2)	15.353(2)
β / °	109.139(10)	109.102(10)	109.157(16)	109.185(16)	110.897(18)
V / Å^3	2619.3(4)	2625.4(4)	2630.6(7)	2635.1(7)	2648.3(8)
ρ_{calc} / g cm^{-3}	1.690	1.686	1.683	1.680	1.671
μ / mm^{-1}	1.942	1.937	1.933	1.930	1.921
$F(000)$	1329.481	1329.481	1329.481	1329.481	1329.481
θ range / °	1.70–25.02	2.12–25.03	1.70–25.03	2.12–25.03	2.20–25.03
Reflections collected	40403	33749	41127	41057	40897
Independent reflections	4628	4638	4647	4650	4681
R_{int}	0.1423	0.1599	0.2393	0.2583	0.1750
Data/restraints/parameters	4628/0/313	4638/0/313	4647/0/313	4650/0/313	4681/0/262
Goodness-of-fit on F^2	1.8496	1.7963	1.4921	1.5810	1.6581
R_1 [$I > 2\sigma(I)$]	0.1079	0.1188	0.1062	0.1200	0.1205
wR_2 [$I > 2\sigma(I)$]	0.2810	0.2982	0.2574	0.2892	0.2996
R_1 (all data)	0.1467	0.1678	0.1738	0.1911	0.1751
wR_2 (all data)	0.2982	0.3183	0.2888	0.3232	0.3196

Table S 10: Crystallographic data and structure-refinement parameters for **1** · IPh from 160 to 230 K.

Parameter	2570245	2570246	2570247	2570248
Temperature / K	160	170	200	230
Space group	$P2_1/n$	$P2_1/n$	$P2_1/n$	$P2_1/n$
a / Å	15.3161(11)	15.3571(5)	15.4065(6)	15.4614(7)
b / Å	12.2026(7)	12.2072(3)	12.2289(3)	12.2589(4)
c / Å	15.4429(11)	15.4611(5)	15.5023(6)	15.5469(7)
β / °	110.918(8)	110.885(4)	110.846(4)	110.864(5)
V / Å^3	2696.0(3)	2708.01(16)	2729.52(18)	2753.5(2)
ρ_{calc} / g cm^{-3}	1.642	1.635	1.125	1.607
μ / mm^{-1}	1.887	1.878	0.719	1.847
$F(000)$	1329.481	1329.481	954.913	1329.481
θ range / °	2.19–25.02	2.18–25.03	2.18–25.02	2.17–25.03
Reflections collected	42395	42884	43400	43523
Independent reflections	4771	4803	4841	4872
R_{int}	0.0558	0.0430	0.0465	0.0461
Data/restraints/parameters	4771/0/272	4803/0/282	4841/0/282	4872/0/282
Goodness-of-fit on F^2	1.0083	1.0079	0.9746	1.0139
R_1 [$I > 2\sigma(I)$]	0.0490	0.0343	0.0322	0.0392
wR_2 [$I > 2\sigma(I)$]	0.1206	0.0681	0.0741	0.1020
R_1 (all data)	0.0674	0.0490	0.0471	0.0559
wR_2 (all data)	0.1297	0.0730	0.0797	0.1095

Table S 11: Crystallographic data and structure-refinement parameters for **1** · py from 95 to 150 K.

CCDC ref. code	2565664	2565665	2565666 K	2565667	2565668
Temperature / K	95	115	135	145	150
Space group	$P2_1/n$	$P2_1/n$	$P2_1/n$	$P2_1/n$	$P2_1/n$
a / Å	14.6890(9)	14.7135(9)	14.7531(9)	14.8047(9)	14.8800(13)
b / Å	11.9899(5)	12.0011(5)	12.0107(6)	12.0082(6)	11.9936(8)
c / Å	14.8537(7)	14.8597(8)	14.8834(8)	14.9005(8)	14.9016(11)
β / °	110.623(6)	110.561(7)	110.619(7)	110.601(7)	110.451(9)
V / Å^3	2448.4(2)	2456.8(3)	2468.3(3)	2479.6(3)	2491.8(4)
ρ_{calc} / g cm^{-3}	1.469	1.464	1.457	1.450	1.443
μ / mm^{-1}	0.815	0.812	0.809	0.805	0.801
$F(000)$	1123.013	1123.013	1123.013	1123.013	1123.013
θ range / °	2.24–26.36	2.24–26.37	2.24–26.37	2.24–26.37	2.24–26.37
Reflections collected	27140	27020	27436	27601	27854
Independent reflections	4997	5013	5036	5060	5097
R_{int}	0.0388	0.0379	0.0368	0.0357	0.0351
Data/restraints/parameters	4997/0/316	5013/0/316	5036/0/316	5060/0/316	5097/0/316
Goodness-of-fit on F^2	1.0518	1.0556	1.0495	1.0578	1.0577
R_1 [$I > 2\sigma(I)$]	0.0387	0.0395	0.0402	0.0425	0.0449
wR_2 [$I > 2\sigma(I)$]	0.0725	0.0758	0.0767	0.0800	0.0829
R_1 (all data)	0.0605	0.0627	0.0634	0.0673	0.0706
wR_2 (all data)	0.0793	0.0836	0.0846	0.0886	0.0919

Table S 12: Crystallographic data and structure-refinement parameters for **1** · py from 155 to 235 K.

CCDC ref. code	2565669	2565670	2565671	2565672	2565673
Temperature / K	155	175	195	215	235
Space group	$P121/n1$	$P121/n1$	$P121/n1$	$P121/n1$	$P121/n1$
a / Å	14.9190(6)	14.9829(6)	15.0146(6)	15.0532(5)	15.0842(7)
b / Å	11.9563(4)	11.9287(4)	11.9342(4)	11.9435(4)	11.9641(4)
c / Å	14.9841(7)	15.1810(6)	15.2467(6)	15.2993(6)	15.3418(7)
β / °	110.392(5)	110.220(4)	110.162(4)	110.128(4)	110.131(5)
V / Å^3	2505.30(19)	2546.04(18)	2564.60(18)	2582.64(17)	2599.6(2)
ρ_{calc} / g cm^{-3}	1.436	1.413	1.402	1.393	1.384
μ / mm^{-1}	0.797	0.784	0.778	0.773	0.768
$F(000)$	1123.013	1123.013	1123.013	1123.013	1123.013
θ range / °	2.24–28.28	2.23–28.28	2.22–28.28	2.22–28.28	2.21–26.37
Reflections collected	52494	53595	54219	54272	56527
Independent reflections	6210	6311	6356	6407	5300
R_{int}	0.0384	0.0338	0.0306	0.0304	0.0442
Data/restraints/parameters	6210/0/339	6311/0/349	6356/0/359	6407/0/354	5300/0/354
Goodness-of-fit on F^2	1.0683	1.0694	1.0632	1.0687	1.0468
R_1 [$I > 2\sigma(I)$]	0.0444	0.0410	0.0356	0.0362	0.0267
wR_2 [$I > 2\sigma(I)$]	0.0771	0.0718	0.0645	0.0648	0.0571
R_1 (all data)	0.0710	0.0662	0.0589	0.0614	0.0435
wR_2 (all data)	0.0857	0.0802	0.0718	0.0728	0.0633

Table S 13: Crystallographic data and structure-refinement parameters for **1** · py at 255 K.

CCDC ref. code	2565674
Temperature / K	255
Space group	$P121/n1$
a / Å	15.1196(10)
b / Å	11.9834(5)
c / Å	15.3909(10)
β / °	110.127(7)
V / Å^3	2618.3(3)
ρ_{calc} / g cm^{-3}	1.374
μ / mm^{-1}	0.762
$F(000)$	1123.013
θ range / °	2.21–26.37
Reflections collected	38477
Independent reflections	5349
R_{int}	0.0447
Data/restraints/parameters	5349/0/354
Goodness-of-fit on F^2	1.0836
R_1 [$I > 2\sigma(I)$]	0.0390
wR_2 [$I > 2\sigma(I)$]	0.0859
R_1 (all data)	0.0638
wR_2 (all data)	0.0948

3 NCI Plots and Hirshfeld Surface Analysis

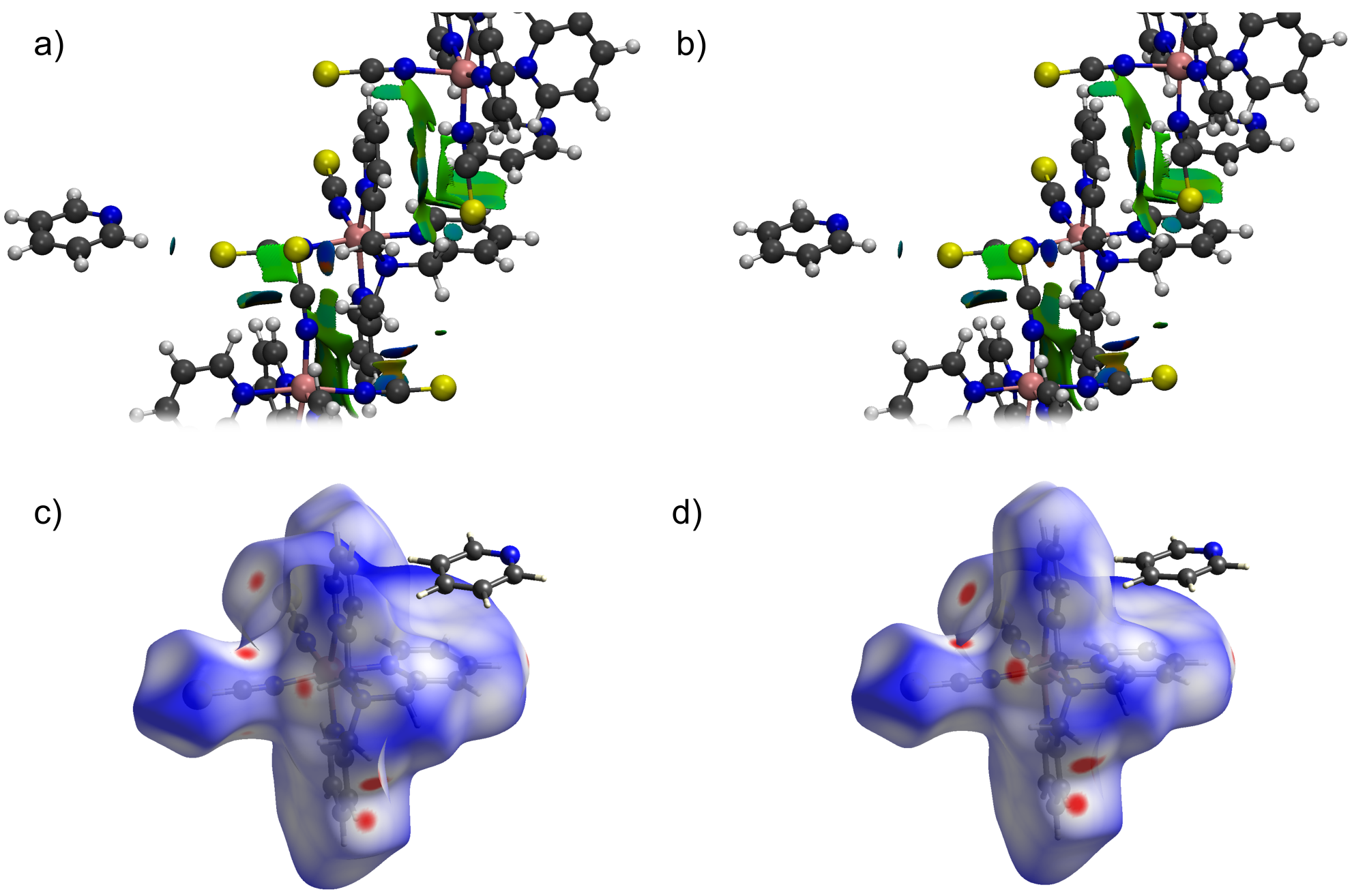

Fig. S6: NCI plots of **1** · py, highlighting the relevant short contacts at 175K (a) and 155K (b), the RDG is cut at 0.4, while sign(λ)ρ is colour-coded from -0.015 a.u. (blue) via 0 (green) to 0.015 a.u. (red). Hirshfeld surface mapped with dnorm at 175K (c) and 155K (d).

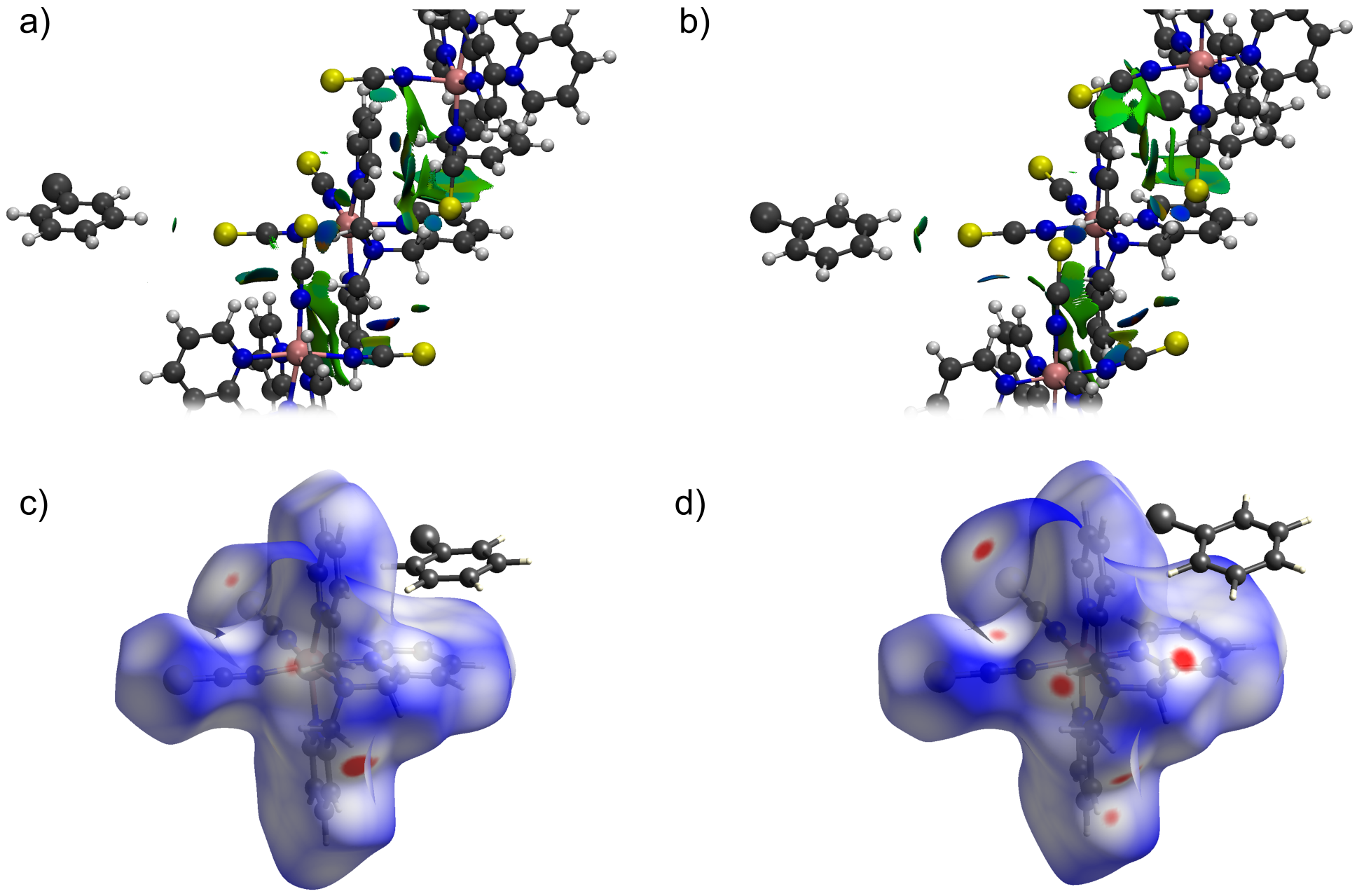

Fig. S7: NCI plots of **1** · ClPh, highlighting the relevant short contacts at 170K (a) and 160K (b), the RDG is cut at 0.4, while sign(λ)ρ is colour-coded from -0.015 a.u. (blue) via 0 (green) to 0.015 a.u. (red). Hirshfeld surface mapped with dnorm at 170K (c) and 160K (d).

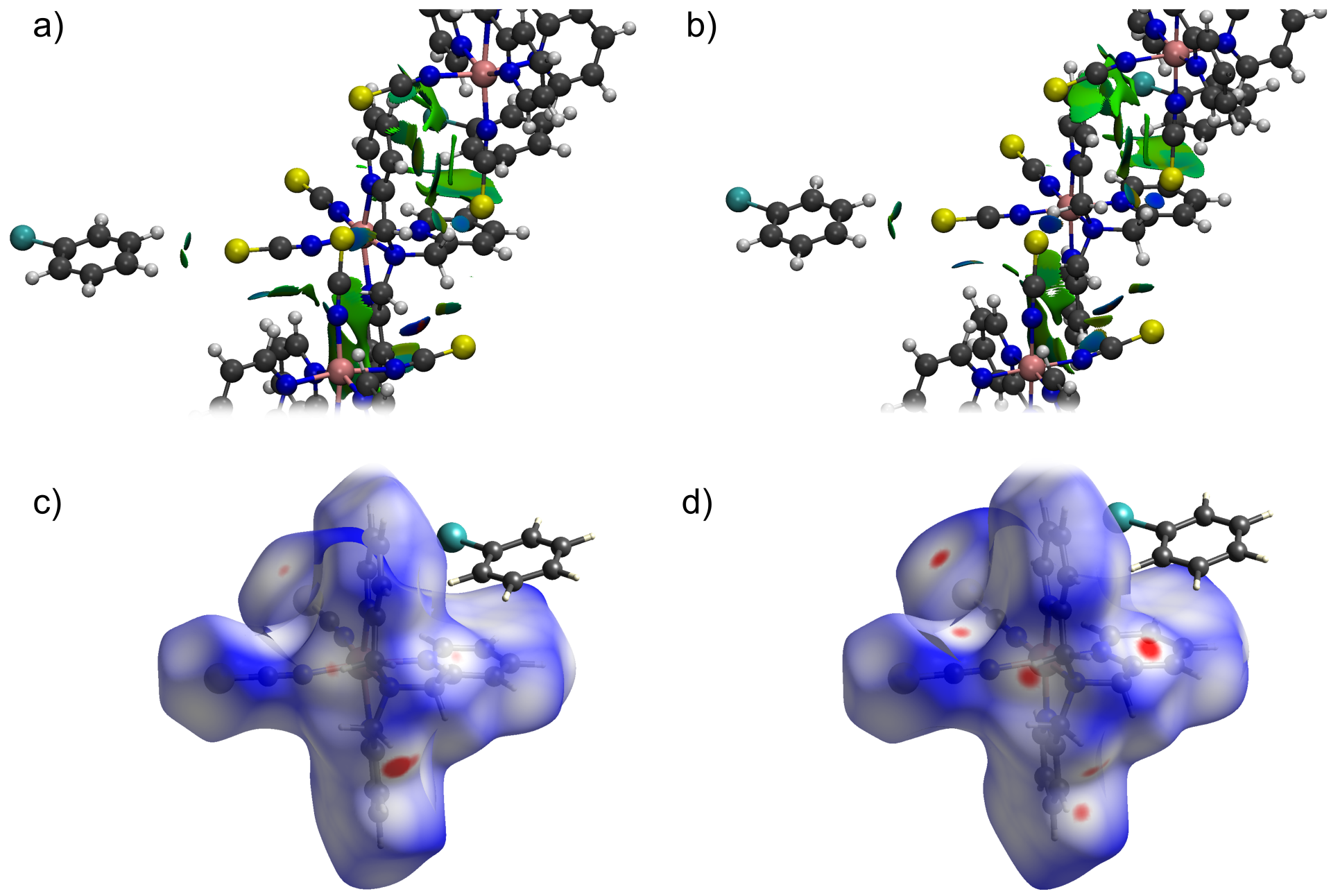

Fig. S8: NCI plots of **1** · BrPh, highlighting the relevant short contacts at 190K (a) and 230K (b), the RDG is cut at 0.4, while sign(λ)ρ is colour-coded from -0.015 a.u. (blue) via 0 (green) to 0.015 a.u. (red). Hirshfeld surface mapped with dnorm at 190K (c) and 230K (d).

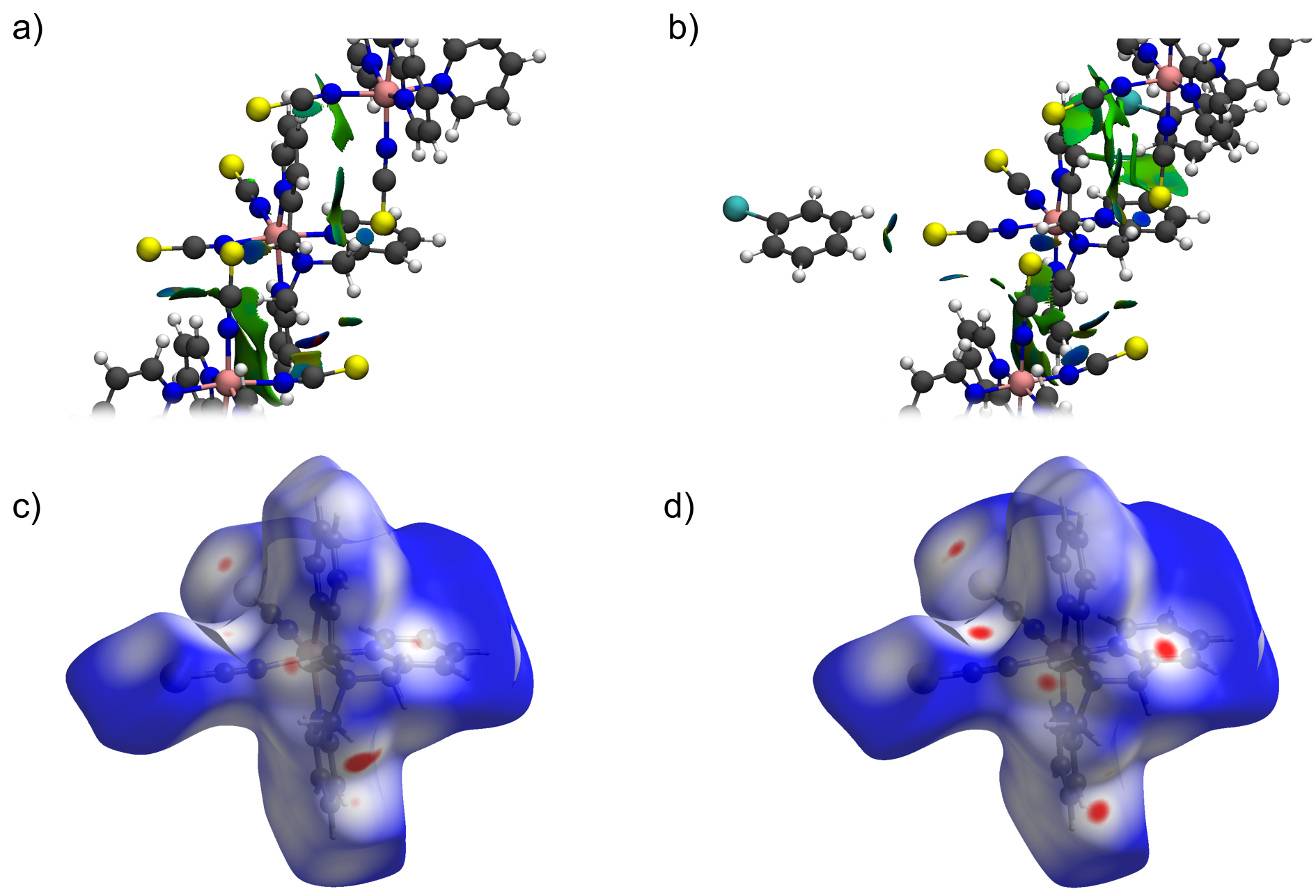

Fig. S9: NCI plots of **1** · IPh, highlighting the relevant short contacts at 155K (a) and 150K (b), the RDG is cut at 0.4, while sign(λ)ρ is colour-coded from -0.015 a.u. (blue) via 0 (green) to 0.015 a.u. (red). Hirshfeld surface mapped with dnorm at 155K (c) and 150K (d).

4 NCI Plot Indices

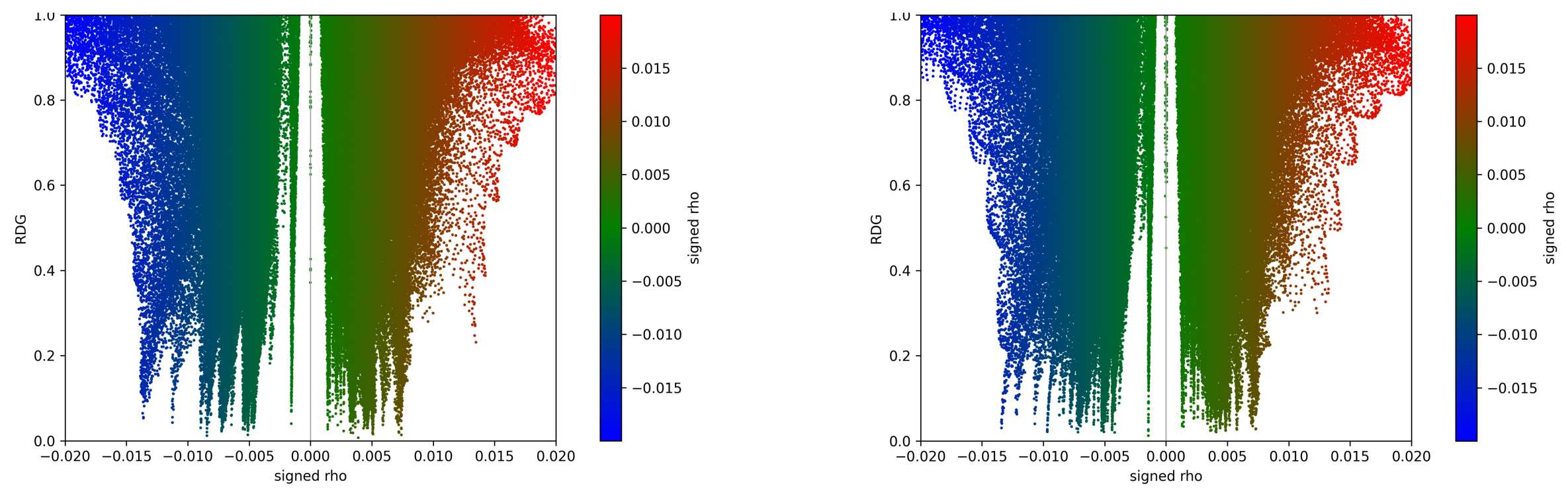

Fig. S10: RDG vs signed rho scatter plots of **1** · py before (left) and after (right) the transition.

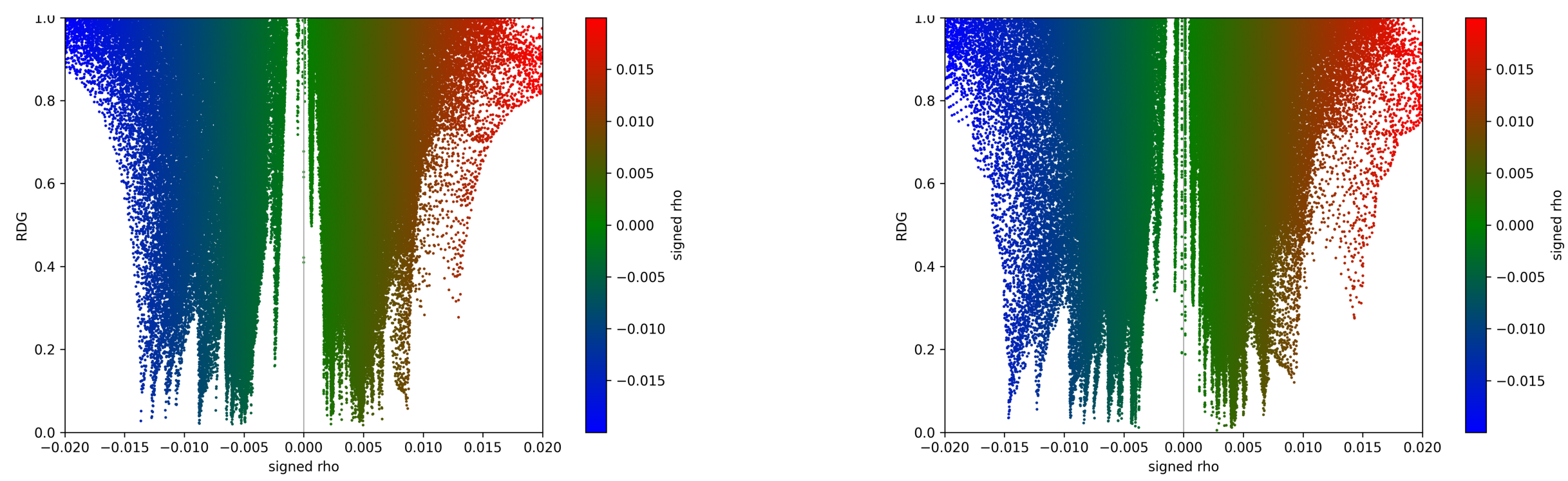

Fig. S11: RDG vs signed rho scatter plots of **1** · FPh before (left) and after (right) the transition.

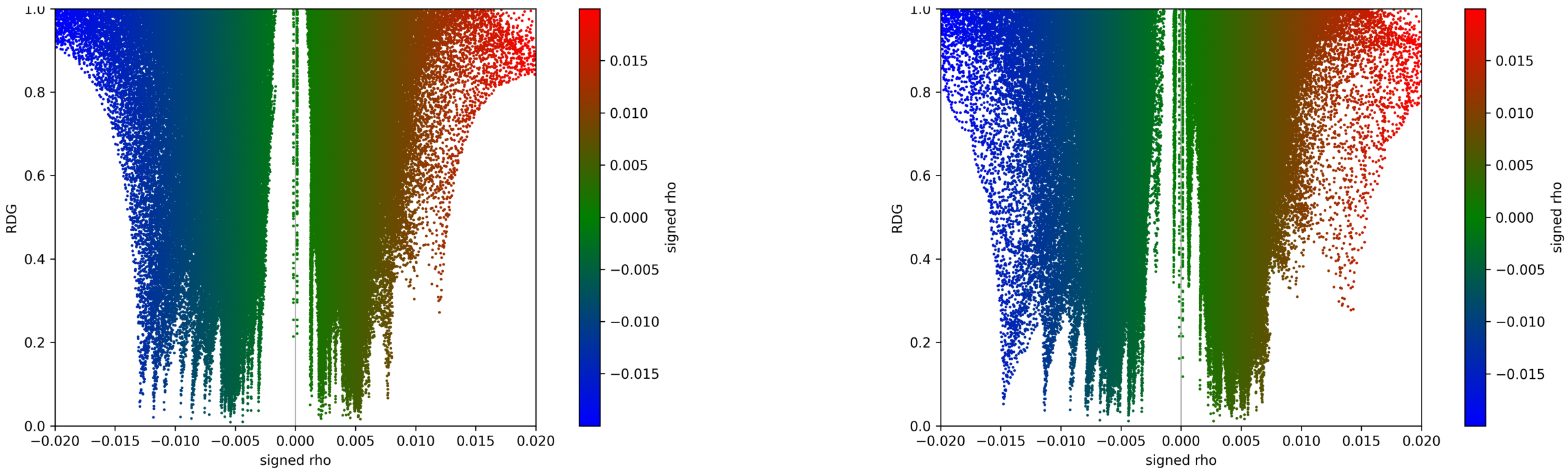

Fig. S12: RDG vs signed rho scatter plots of **1** · ClPh before (left) and after (right) the transition.

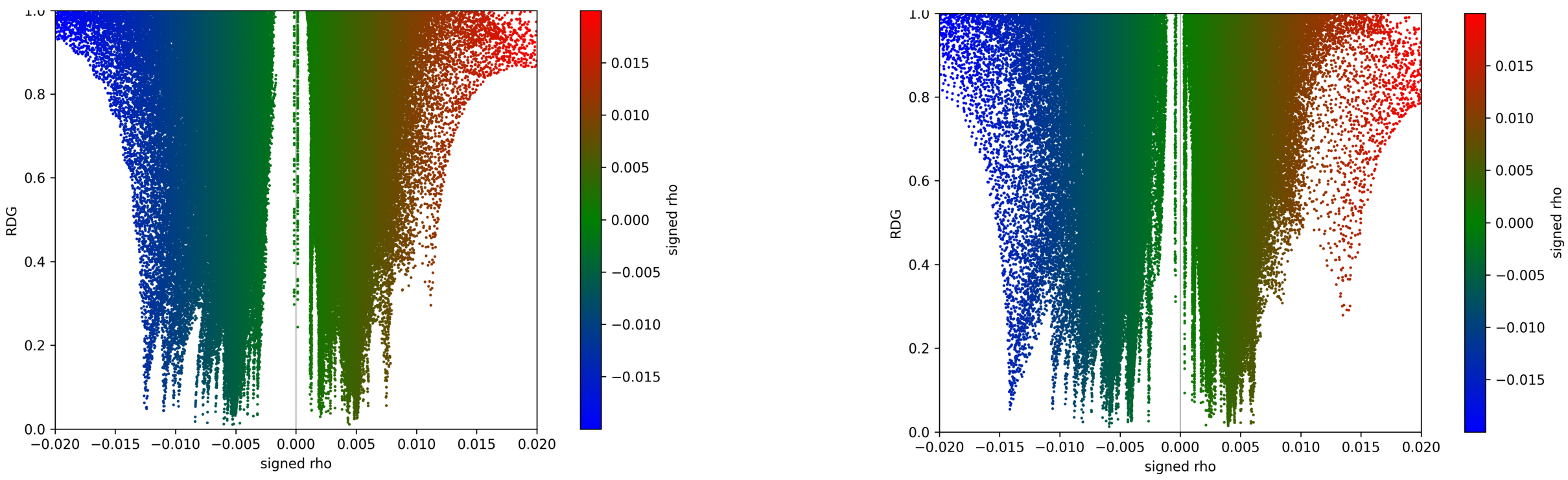

Fig. S13: RDG vs signed rho scatter plots of **1** · BrPh before (left) and after (right) the transition.

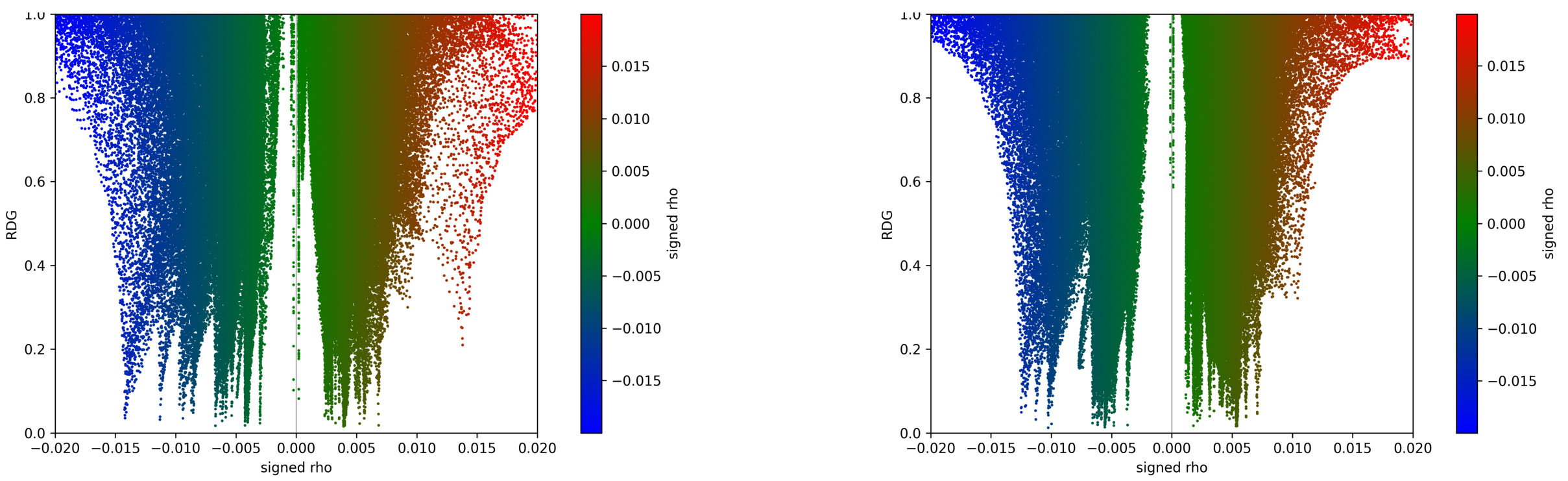

Fig. S14: RDG vs signed rho scatter plots of **1** · IPh before (left) and after (right) the transition.

5 2D Fingerprint Plots

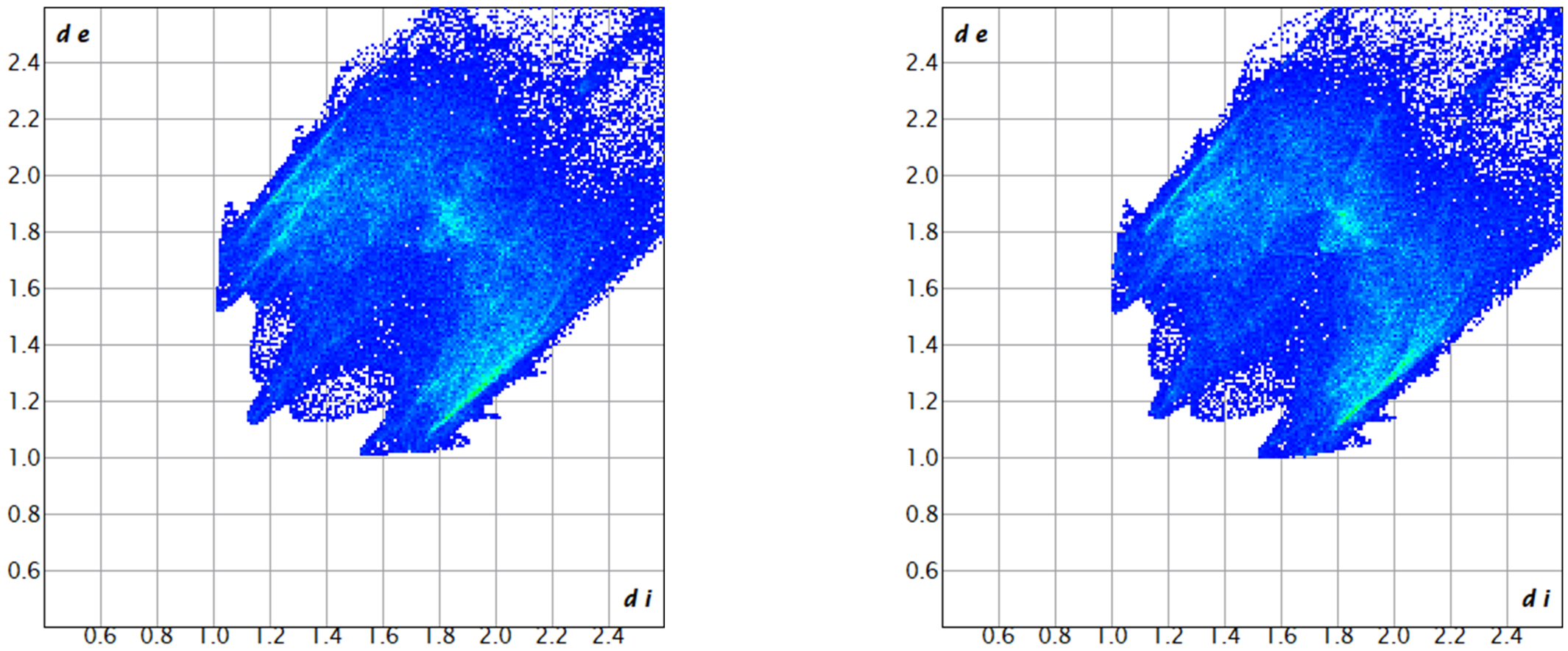

Fig. S15: Fingerprint plot of **1** · py before (left) and after (right) the transition.

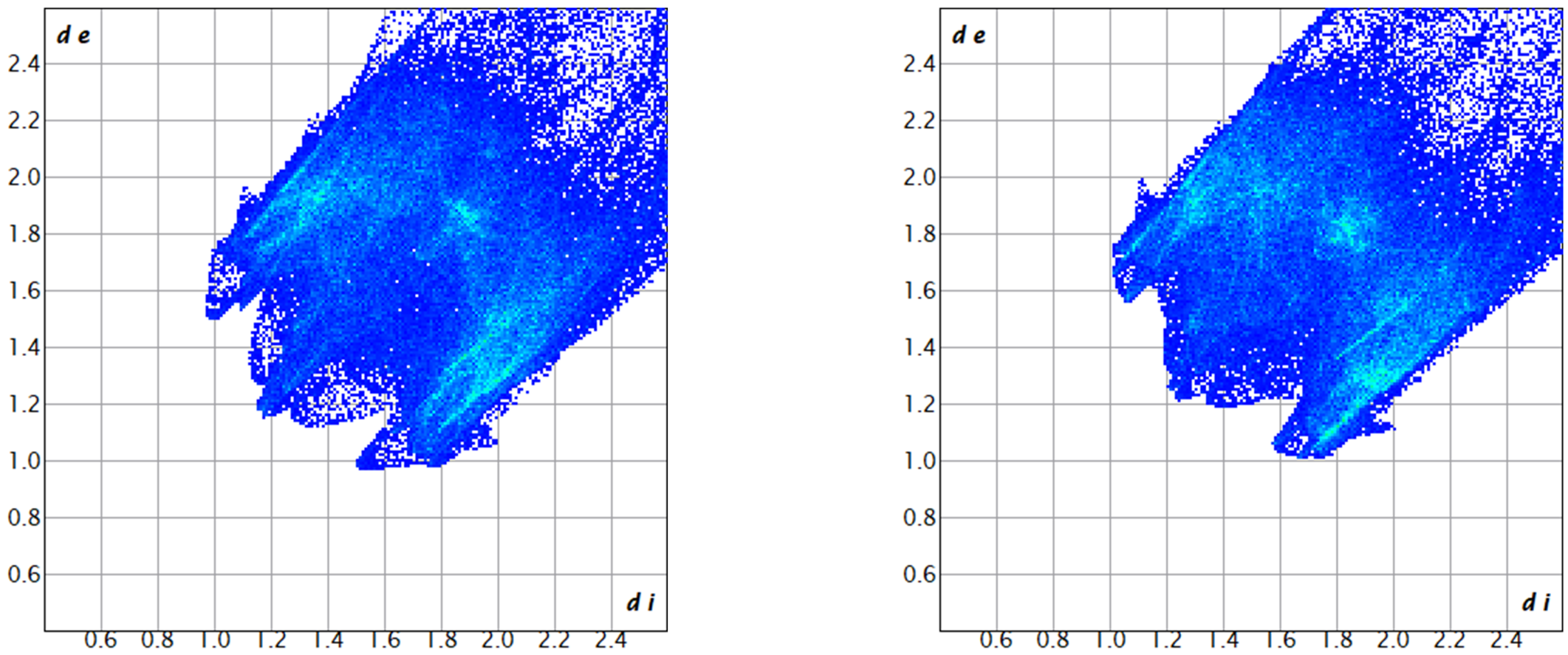

Fig. S16: Fingerprint plot of **1** · FPh before (left) and after (right) the transition.

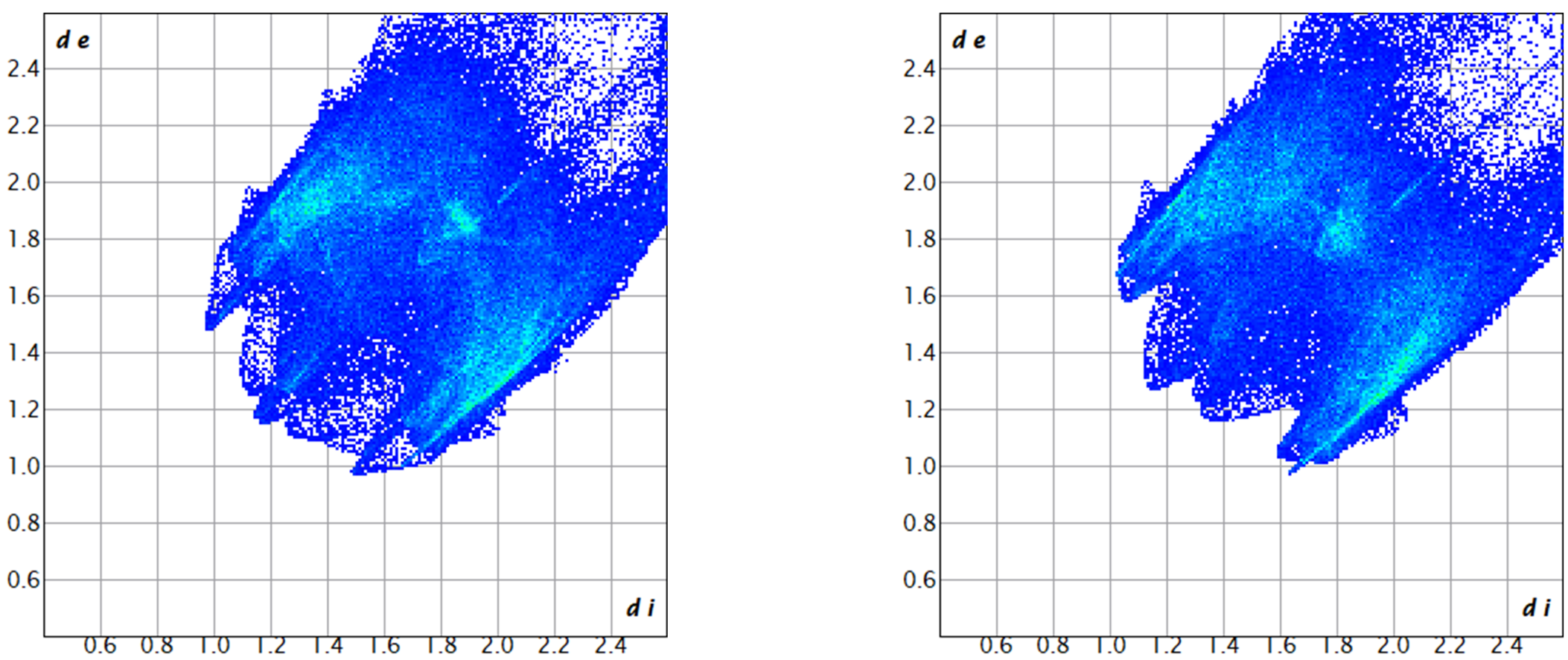

Fig. S17: Fingerprint plot of **1** · ClPh before (left) and after (right) the transition.

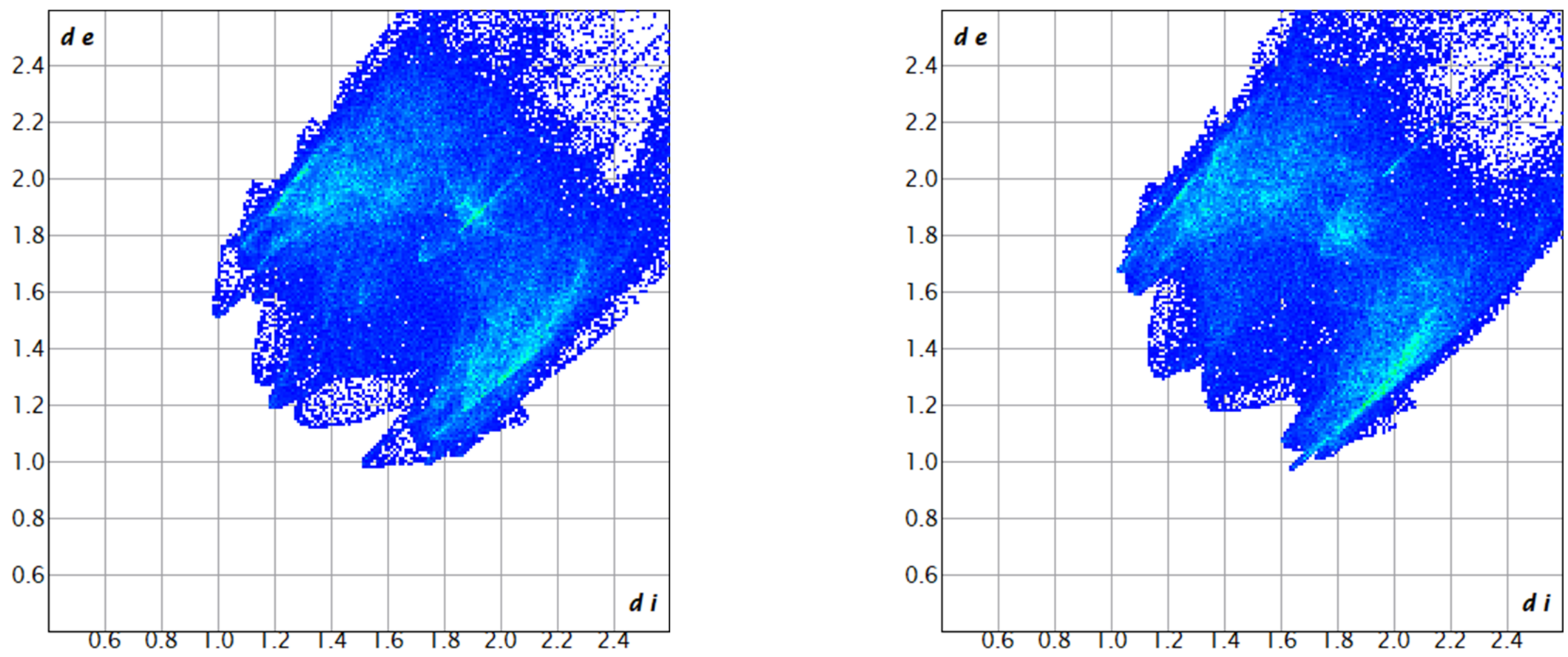

Fig. S18: Fingerprint plot of **1** · BrPh before (left) and after (right) the transition.

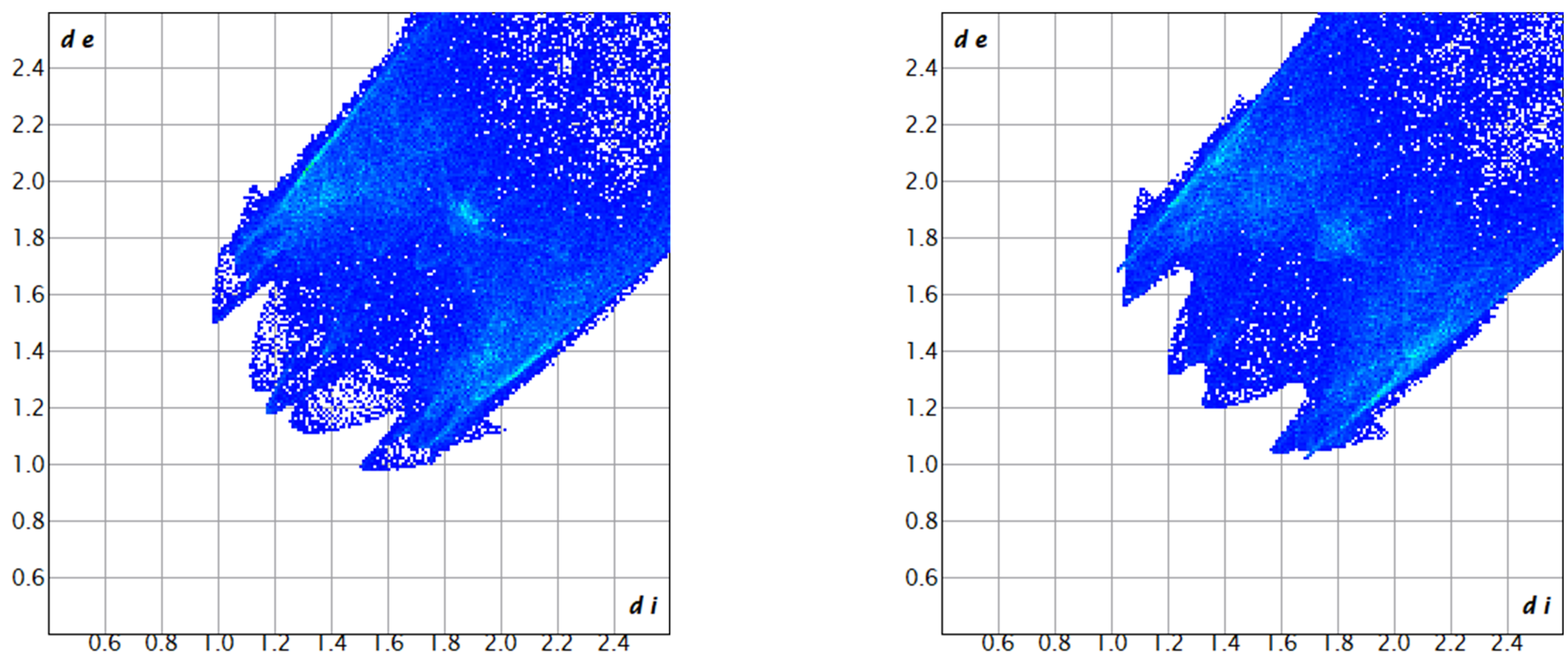

Fig. S19: Fingerprint plot of **1** · IPh before (left) and after (right) the transition.

6 Pairwise Interaction Energies Involving the Solvent Guest

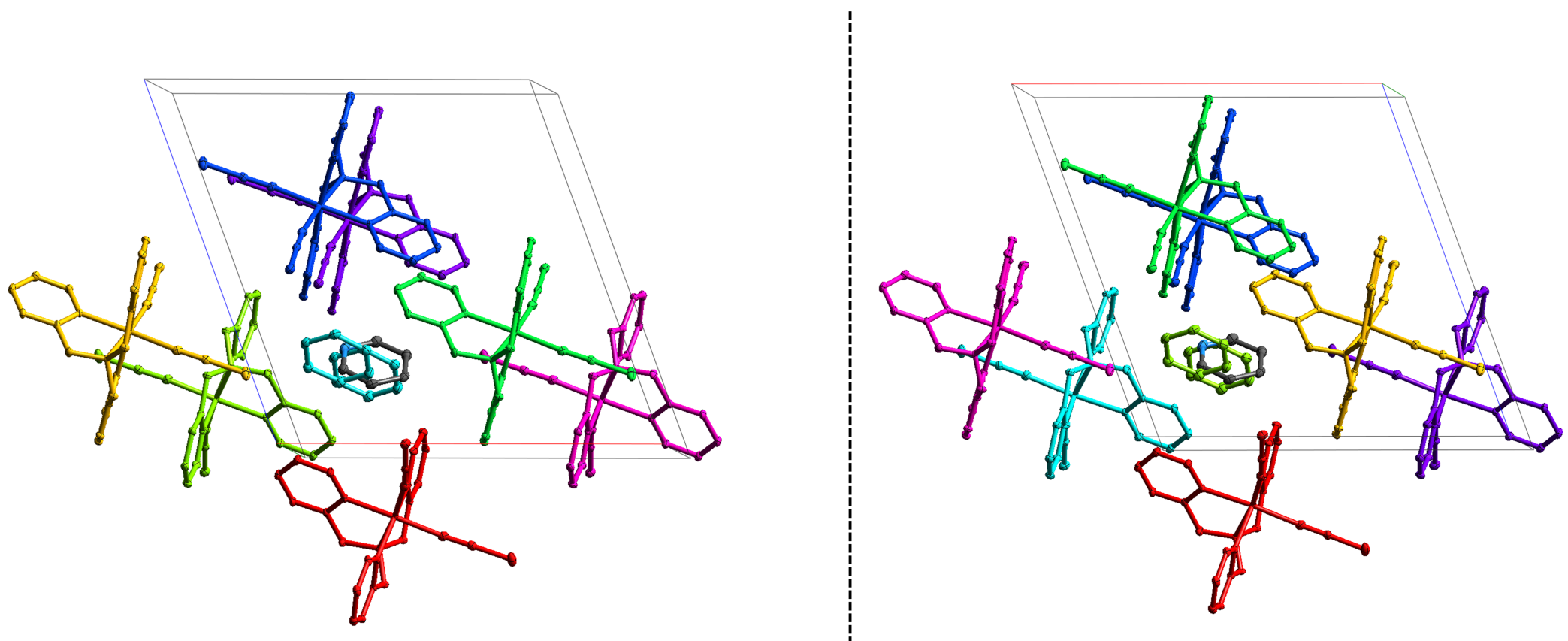

	N	Symop	R	Electron Density	E_ele	E_pol	E_dis	E_rep	E_tot
	1	-	5.98	B3LYP/6-31G(d,p)	-18.3	-8.5	-26.9	28.4	-31.6
	1	-	9.76	B3LYP/6-31G(d,p)	2.6	-2.2	-3.5	5.1	1.2
	1	-	7.54	B3LYP/6-31G(d,p)	-10.9	-3.1	-12.0	7.0	-19.9
	1	-	5.91	B3LYP/6-31G(d,p)	-3.0	-1.7	-36.5	21.0	-23.3
	2	-x+1/2, y+1/2, -z+1/2	6.04	B3LYP/6-31G(d,p)	-0.5	-0.3	-4.8	2.6	-3.4
	1	-	8.07	B3LYP/6-31G(d,p)	-6.5	-1.6	-10.4	7.6	-12.4
	1	-	8.88	B3LYP/6-31G(d,p)	6.6	-3.7	-6.0	9.3	4.8
	1	-	9.54	B3LYP/6-31G(d,p)	-4.1	-2.4	-3.0	2.7	-7.1

	N	Symop	R	Electron Density	E_ele	E_pol	E_dis	E_rep	E_tot
	1	-	5.99	B3LYP/6-31G(d,p)	-17.0	-8.3	-25.9	25.0	-31.3
	1	-	5.93	B3LYP/6-31G(d,p)	-2.6	-1.8	-35.4	19.1	-23.1
	2	-x+1/2, y+1/2, -z+1/2	6.04	B3LYP/6-31G(d,p)	-0.6	-0.3	-4.9	2.6	-3.4
	1	-	8.17	B3LYP/6-31G(d,p)	-6.5	-1.6	-9.8	6.8	-12.5
	1	-	7.64	B3LYP/6-31G(d,p)	-10.5	-2.9	-11.0	6.0	-19.1
	1	-	8.96	B3LYP/6-31G(d,p)	7.5	-3.7	-5.8	8.5	5.4
	1	-	9.52	B3LYP/6-31G(d,p)	-4.6	-2.7	-3.4	3.7	-7.5
	1	-	9.80	B3LYP/6-31G(d,p)	2.5	-2.3	-3.6	5.5	1.3

Fig. S20: Crystal Explorer output of the pairwise interaction energies involving the pyridine guest molecule at 175K (right) and 155K (left). Note: Colorschemes are arbitrarily chosen by CE and do no not match between the two temperatures.

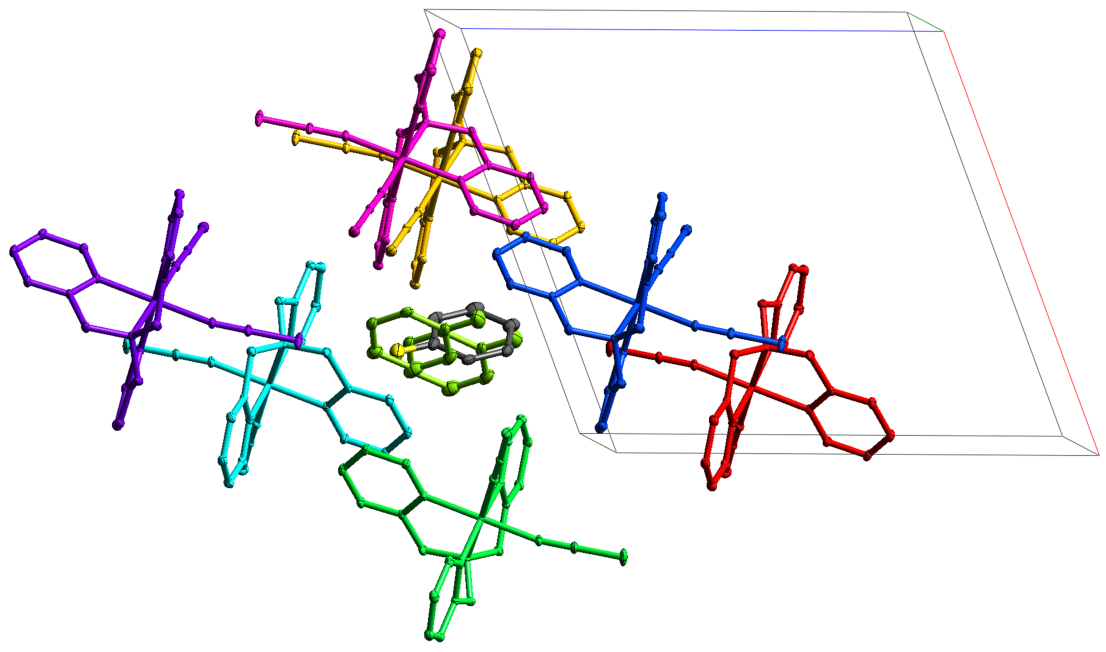

	N	Symop	R	Electron Density	E_ele	E_pol	E_dis	E_rep	E_tot
	1	-	10.13	B3LYP/6-31G(d,p)	-1.7	-1.6	-1.9	0.4	-4.3
	1	-	8.78	B3LYP/6-31G(d,p)	-6.7	-4.3	-7.4	12.3	-9.1
	2	-x+1/2, y+1/2, -z+1/2	6.29	B3LYP/6-31G(d,p)	0.3	-0.1	-4.1	0.9	-2.8
	1	-	5.74	B3LYP/6-31G(d,p)	-22.1	-9.0	-32.1	34.6	-36.6
	1	-	7.79	B3LYP/6-31G(d,p)	-2.6	-1.8	-10.7	5.3	-10.1
	1	-	6.21	B3LYP/6-31G(d,p)	-5.9	-1.4	-41.0	29.1	-25.1
	1	-	9.81	B3LYP/6-31G(d,p)	3.3	-1.7	-3.4	4.2	1.8
	1	-	7.94	B3LYP/6-31G(d,p)	-2.3	-0.8	-11.9	6.1	-9.6

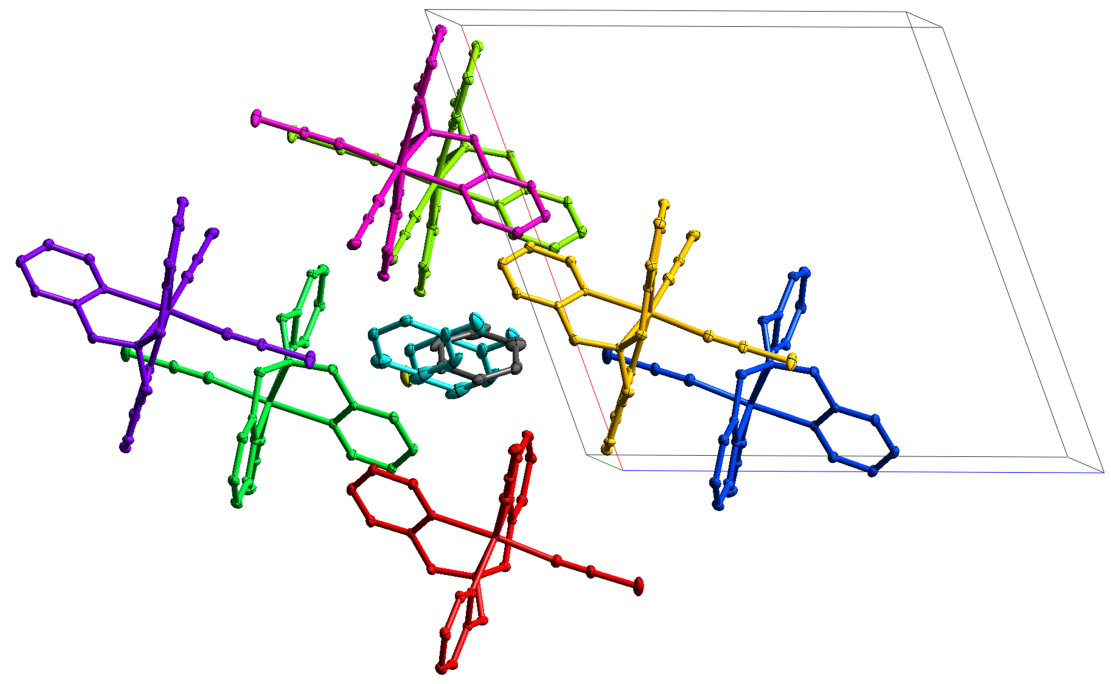

	N	Symop	R	Electron Density	E_ele	E_pol	E_dis	E_rep	E_tot
	1	-	5.79	B3LYP/6-31G(d,p)	-20.0	-8.8	-30.5	30.6	-35.3
	1	-	6.20	B3LYP/6-31G(d,p)	-5.6	-1.6	-40.1	26.1	-25.9
	1	-	9.05	B3LYP/6-31G(d,p)	-4.6	-4.0	-6.3	7.6	-8.6
	1	-	7.91	B3LYP/6-31G(d,p)	-2.2	-1.7	-10.1	5.0	-9.3
	2	-x+1/2, y+1/2, -z+1/2	6.05	B3LYP/6-31G(d,p)	-0.0	-0.2	-6.0	2.2	-4.0
	1	-	10.01	B3LYP/6-31G(d,p)	-2.3	-2.3	-2.8	1.9	-5.4
	1	-	9.86	B3LYP/6-31G(d,p)	1.9	-1.9	-3.6	4.6	0.3
	1	-	8.12	B3LYP/6-31G(d,p)	-1.7	-0.9	-11.1	6.5	-8.2

Fig. S21: Crystal Explorer output of the pairwise interaction energies involving the fluorobenzene guest molecule at 175K (right) and 155K (left). Note: Colorschemes are arbitrarily chosen by CE and do no not match between the two temperatures.

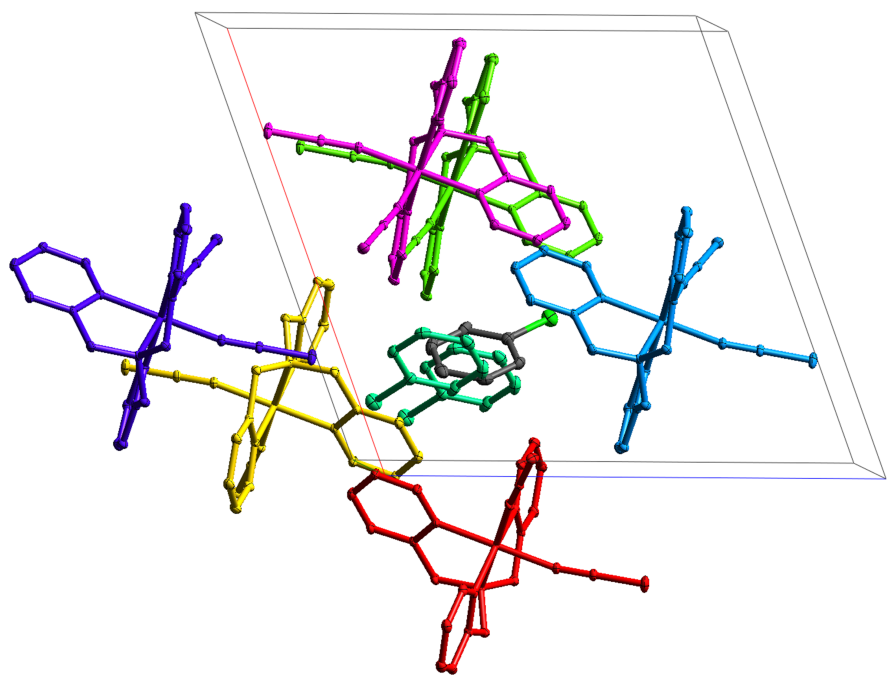

	N	Symop	R	Electron Density	E_ele	E_pol	E_dis	E_rep	E_tot
	1	-	6.14	B3LYP/6-31G(d,p)	-19.6	-8.3	-31.4	33.5	-33.5
	1	-	8.23	B3LYP/6-31G(d,p)	0.8	-2.0	-11.3	5.9	-6.9
	1	-	8.06	B3LYP/6-31G(d,p)	-17.7	-5.6	-8.1	22.1	-16.3
	2	-x+1/2, y+1/2, -z+1/2	6.73	B3LYP/6-31G(d,p)	-1.3	-0.3	-6.0	3.3	-4.7
	1	-	5.71	B3LYP/6-31G(d,p)	-4.4	-1.8	-41.9	29.4	-24.3
	1	-	10.47	B3LYP/6-31G(d,p)	-7.6	-2.8	-4.6	4.1	-11.6
	1	-	8.48	B3LYP/6-31G(d,p)	-1.9	-1.0	-11.1	7.6	-7.8

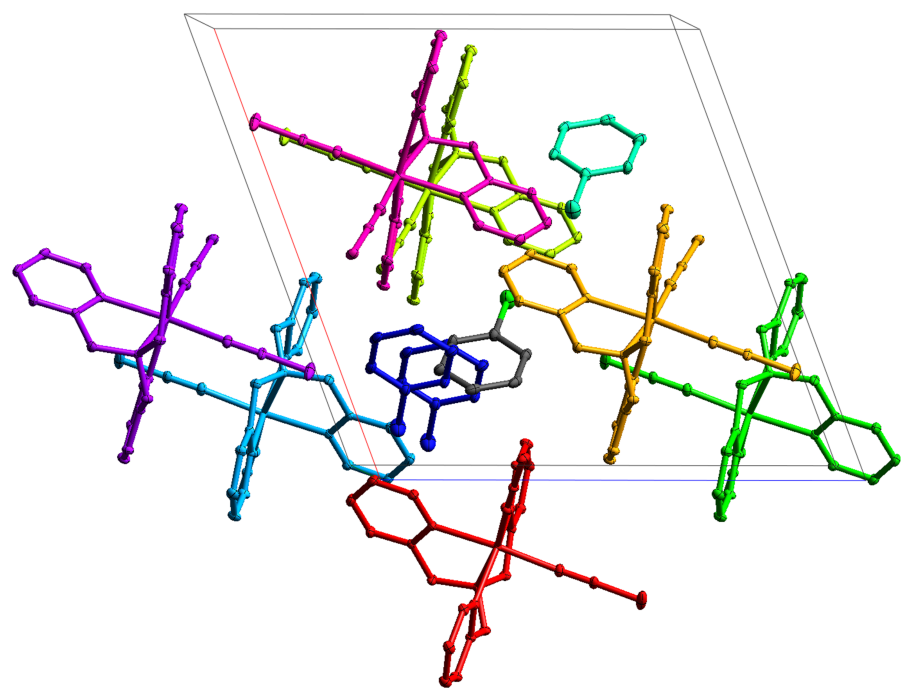

	N	Symop	R	Electron Density	E_ele	E_pol	E_dis	E_rep	E_tot
	1	-	6.57	B3LYP/6-31G(d,p)	-14.5	-8.5	-29.0	22.8	-32.8
	1	-	5.75	B3LYP/6-31G(d,p)	-5.8	-2.1	-47.7	36.2	-26.8
	1	-	7.95	B3LYP/6-31G(d,p)	-11.0	-5.8	-11.3	20.5	-13.1
	1	-	9.07	B3LYP/6-31G(d,p)	3.1	-3.1	-4.5	3.6	-0.7
	1	-x, -y, -z	7.09	B3LYP/6-31G(d,p)	-1.0	-0.2	-3.8	6.8	-0.2
	1	-	8.30	B3LYP/6-31G(d,p)	0.6	-1.6	-8.1	2.5	-6.1
	2	-x+1/2, y+1/2, -z+1/2	6.53	B3LYP/6-31G(d,p)	-3.4	-0.4	-7.1	7.1	-5.7
	1	-	10.69	B3LYP/6-31G(d,p)	-4.7	-1.8	-2.3	1.3	-7.5
	1	-	8.44	B3LYP/6-31G(d,p)	-1.0	-0.9	-9.3	3.9	-7.4

Fig. S22: Crystal Explorer output of the pairwise interaction energies involving the chlorobenzene guest molecule at 175K (right) and 155K (left). Note: Colorschemes are arbitrarily chosen by CE and do no not match between the two temperatures.

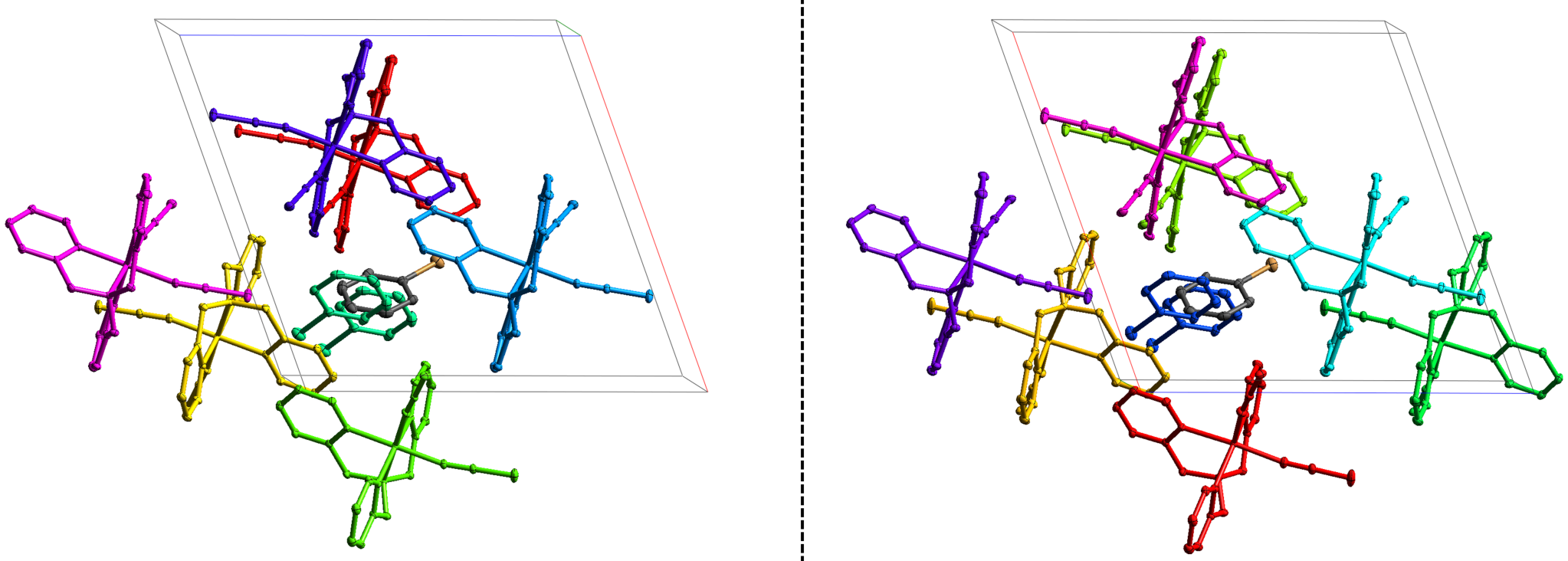

	N	Symop	R	Electron Density	E_ele	E_pol	E_dis	E_rep	E_tot
	1	-	7.71	B3LYP/6-31G(d,p)	-16.8	-5.6	-9.0	21.4	-16.6
	1	-	8.48	B3LYP/6-31G(d,p)	0.9	-2.0	-11.5	5.7	-7.1
	1	-	6.47	B3LYP/6-31G(d,p)	-19.6	-8.4	-32.1	33.5	-34.2
	2	-x+1/2, y+1/2, -z+1/2	7.14	B3LYP/6-31G(d,p)	-2.5	-0.4	-7.3	6.5	-5.3
	1	-	5.58	B3LYP/6-31G(d,p)	-5.7	-1.9	-43.5	30.5	-26.4
	1	-	8.77	B3LYP/6-31G(d,p)	-2.0	-1.0	-11.1	7.1	-8.2
	1	-	10.95	B3LYP/6-31G(d,p)	-8.0	-2.9	-4.9	4.8	-11.9

	N	Symop	R	Electron Density	E_ele	E_pol	E_dis	E_rep	E_tot
	1	-	6.58	B3LYP/6-31G(d,p)	-16.4	-8.1	-29.9	25.4	-33.6
	1	-	8.73	B3LYP/6-31G(d,p)	1.3	-1.9	-9.8	4.2	-5.9
	1	-	7.75	B3LYP/6-31G(d,p)	-16.1	-6.4	-10.2	18.5	-19.3
	1	-	8.77	B3LYP/6-31G(d,p)	3.1	-3.1	-4.9	12.5	4.3
	1	-	5.49	B3LYP/6-31G(d,p)	-6.0	-2.2	-45.1	32.1	-27.4
	2	-x+1/2, y+1/2, -z+1/2	7.03	B3LYP/6-31G(d,p)	-3.5	-0.5	-8.5	9.2	-5.9
	1	-	11.17	B3LYP/6-31G(d,p)	-7.5	-2.7	-4.4	4.1	-11.2
	1	-	8.84	B3LYP/6-31G(d,p)	-1.8	-1.1	-11.1	6.5	-8.3

Fig. S23: Crystal Explorer output of the pairwise interaction energies involving the bromobenzene guest molecule at 175K (right) and 155K (left). Note: Colorschemes are arbitrarily chosen by CE and do no not match between the two temperatures.

7 Determination of the Pyridine Orientation

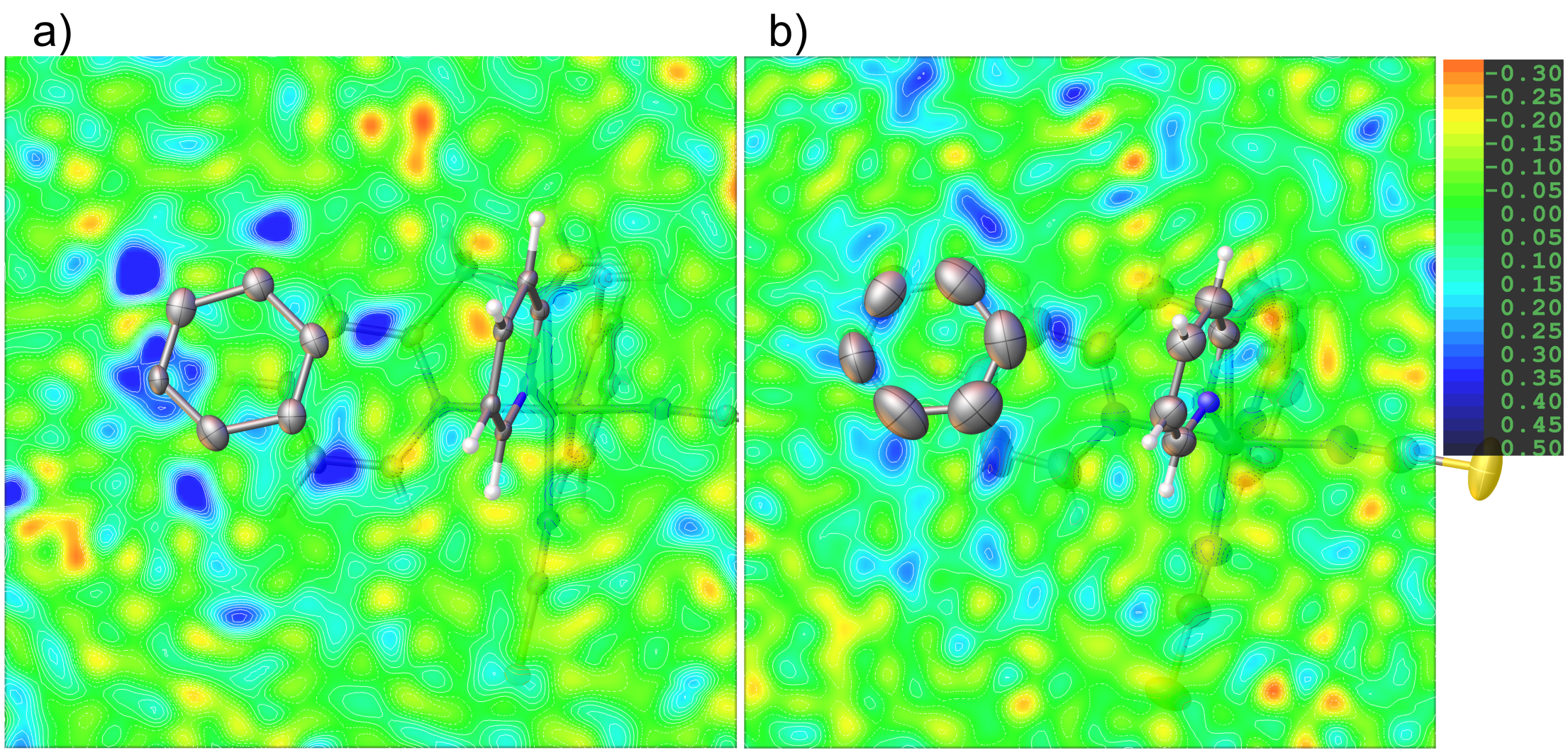

Fig. S24: Difference Fourier maps along the pyridine plane (scale in electrons/Å^3), the missing hydrogen atoms are evident at both 95K (a) and 355K (b). Also the position of the nitrogen site at the misplaced atom site is visible at both temperatures.

8 Intermolecular Interactions along the a-Axis

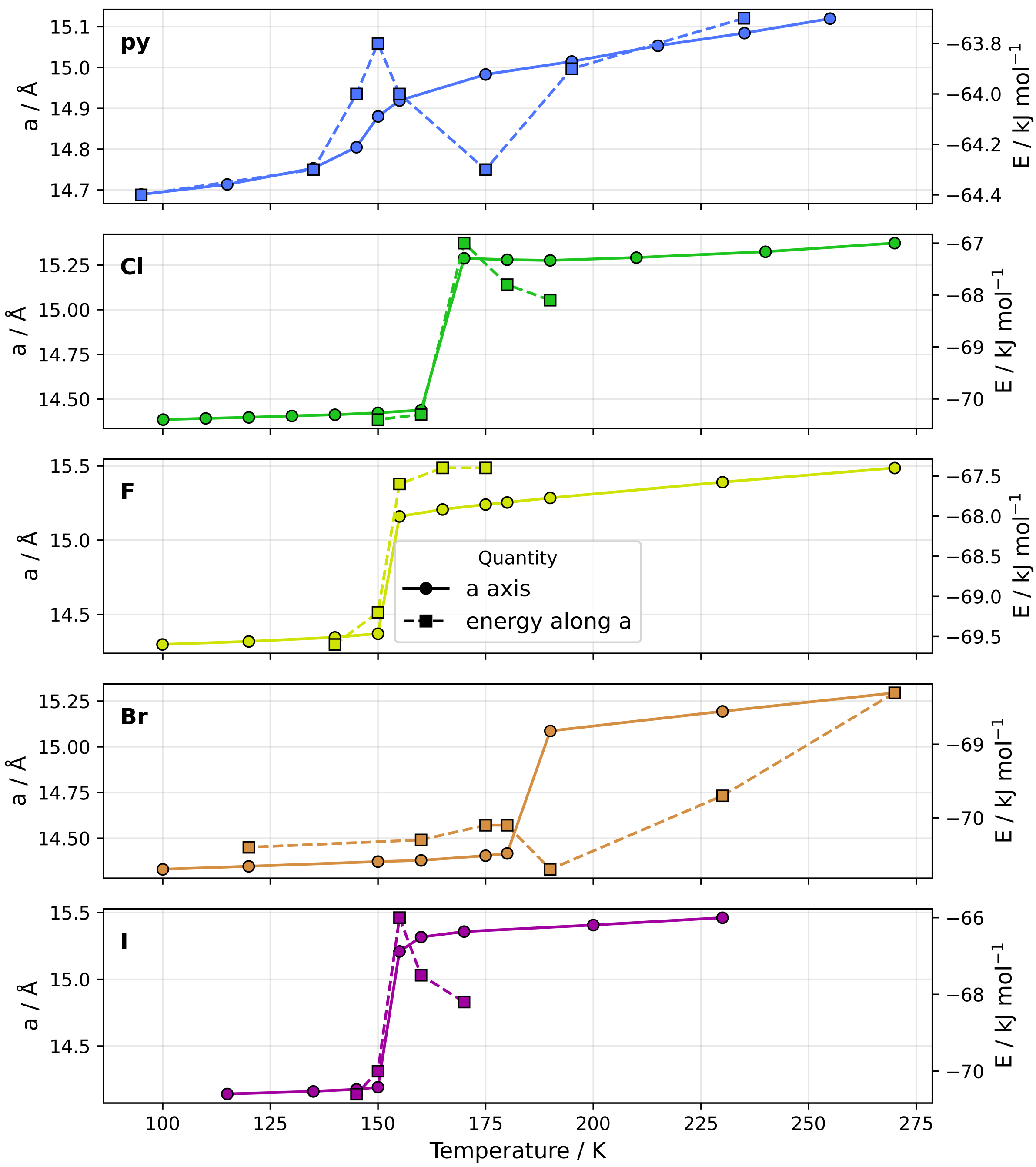

Fig. S25: Temperature dependence of the a-unit cell parameter (circles) together with the pairwise interaction energy of 1 (squares) with its next neighbour generated by the x+$\frac{1}{2}$, -y+1/2, z+1/2 sym. operation. Errors on a are shown but hidden behind the marker.

9 References

(1) Li, B., Wei, R.-J., Tao, J., Huang, R.-B., Zheng, L.-S. and Zheng, Z. (2010). *J. Am. Chem. Soc.* **132**, 1558–1566.
